# Origin and diversification of *Xanthomonas citri* subsp. *citri* pathotypes revealed by inclusive phylogenomic, dating, and biogeographic analyses

**DOI:** 10.1186/s12864-019-6007-4

**Published:** 2019-09-09

**Authors:** José S. L. Patané, Joaquim Martins, Luiz Thiberio Rangel, José Belasque, Luciano A. Digiampietri, Agda Paula Facincani, Rafael Marini Ferreira, Fabrício José Jaciani, Yunzeng Zhang, Alessandro M. Varani, Nalvo F. Almeida, Nian Wang, Jesus A. Ferro, Leandro M. Moreira, João C. Setubal

**Affiliations:** 1 Departamento de Bioquímica, Instituto de Química, Universidade de São Paulo, São Paulo, SP Brazil; 2 Laboratório Especial de Ciclo Celular, Instituto Butantan, São Paulo, SP Brazil; 3 Departamento de Fitopatologia e Nematologia, Escola Superior de Agricultura “Luiz de Queiroz”, Universidade de São Paulo, Piracicaba, SP Brazil; 4 Escola de Artes, Ciências e Humanidades, Universidade de São Paulo, São Paulo, SP Brazil; 5 Faculdade de Ciências Agrárias e Veterinárias, Universidade Estadual Paulista (UNESP), Jaboticabal, SP Brazil; 6 Departamento de Pesquisa e Desenvolvimento, Fundo de Defesa da Citricultura (Fundecitrus), Araraquara, SP Brazil; 7 Citrus Research and Education Center, Department of Microbiology and Cell Science, University of Florida, Lake Alfred, FL USA; 8 Faculdade de Computação, Universidade Federal de Mato Grosso do Sul, Campo Grande, MS Brazil; 9 Núcleo de Pesquisas em Ciências Biológicas, Universidade Federal de Ouro Preto, Ouro Preto, MG Brazil; 10 Biocomplexity Institute of Virginia Tech, Blacksburg, VA USA

**Keywords:** Phylogenomics, Genome evolution, Recombination, Divergence dating, Biogeography

## Abstract

**Background:**

*Xanthomonas citri* subsp. *citri* pathotypes cause bacterial citrus canker, being responsible for severe agricultural losses worldwide. The A pathotype has a broad host spectrum, while A* and A^w^ are more restricted both in hosts and in geography. Two previous phylogenomic studies led to contrasting well-supported clades for sequenced genomes of these pathotypes. No extensive biogeographical or divergence dating analytic approaches have been so far applied to available genomes.

**Results:**

Based on a larger sampling of genomes than in previous studies (including six new genomes sequenced by our group, adding to a total of 95 genomes), phylogenomic analyses resulted in different resolutions, though overall indicating that A + A^W^ is the most likely true clade. Our results suggest the high degree of recombination at some branches and the fast diversification of lineages are probable causes for this phylogenetic blurring effect. One of the genomes analyzed, *X. campestris* pv. *durantae*, was shown to be an A* strain; this strain has been reported to infect a plant of the family Verbenaceae, though there are no reports of any *X. citri* subsp. *citri* pathotypes infecting any plant outside the Citrus genus. Host reconstruction indicated the pathotype ancestor likely had plant hosts in the family Fabaceae, implying an ancient jump to the current Rutaceae hosts. Extensive dating analyses indicated that the origin of *X. citri* subsp. *citri* occurred more recently than the main phylogenetic splits of Citrus plants, suggesting dispersion rather than host-directed vicariance as the main driver of geographic expansion. An analysis of 120 pathogenic-related genes revealed pathotype-associated patterns of presence/absence.

**Conclusions:**

Our results provide novel insights into the evolutionary history of *X. citri* subsp. *citri* as well as a sound phylogenetic foundation for future evolutionary and genomic studies of its pathotypes.

**Electronic supplementary material:**

The online version of this article (10.1186/s12864-019-6007-4) contains supplementary material, which is available to authorized users.

## Background

Citrus canker is a bacterial disease affecting all commercial citrus varieties. This disease has been intensively studied in the past several decades, given the widespread cultivation of citrus in many regions of the world and the economic importance of the orange juice industry [1–3]. Citrus canker is usually classified into three types: A, B, and C. Type A is believed to have originated in Asia, probably in Southern China, Indonesia, or India, being the most widespread and causing the greatest economic damage [4–6]; it was first recorded in India, around 1830 [7]. Type B (or false canker) was originally identified in Argentina in 1923, and is currently known to be present only in Argentina, Paraguay, and Uruguay [1], whereas type C is limited to the state of São Paulo, Brazil [8]. Types B and C are considered attenuated forms of type A. The causal agent of canker A is *Xanthomonas citri* subsp. *citri* (XCC), which was also the first *Xanthomonas* genome to be sequenced (strain 306) [9].

Two variant forms of citrus canker A are currently known. One is XCC variant A*, and was first found in Southeast Asia around the 1990s infecting *C. aurantifolia* [10]; subsequently it was found in Ethiopia [11]. Its host range has been described as restricted to Mexican lime (*Citrus aurantifolia*), Tahiti lime (*C. latifolia*), and alemow (*C. macrophylla*), but not infecting grapefruit (*C. paradisi*). The second variant is known as A^w^ and was first isolated in 2003 in the USA (Southern Florida), infecting *C. aurantifolia* and *C. macrophylla* (alemow) [12]. In this work we refer to A, A*, and A^w^ as *pathotypes* of XCC, following previous studies [13, 14].

Although much has been learned about XCC genomics, their evolutionary history still contains open questions. One of these is the precise evolutionary relationship between the three pathotypes A, A^w^, and A*. Sun, Stall et al. [12] found that clustering based on two restriction endonucleases (*Xba*I and *Spe*I) led to two different resolutions: for *Xba*I, some A strains clustered with A*, and one other A strain clustered with A^w^, while for *Spe*I, A strains clustered with A* strains. Later, AFLP and MLSA based on four housekeeping genes [15] suggested that A* and A^w^ strains were more related than any of them were to A strains, and the authors suggested A^w^ as a junior synonym of A*. Subsequently, Pruvost et al. [16] identified four major clusters based on a categorical minimum spanning tree using an MLVA based on 31 minisatellites, in which A^w^ and A* strains are clearly separated from each other.

More recently, using more inclusive genomic data provided by WGS techniques, Zhang et al. [13] found that [A^w^ + A*] formed a clade separate from pathotype A (so the two lineages with restricted host ranges gathered together). However, Gordon et al. [14] found rather that a [A + A^w^] clade was separated from A* and that the previous result by Zhang et al. [13] was probably due to recombining regions inducing phylogenetic noise, and suggesting that the generalist lineage A evolved more recently from an ancestral population with restricted host range. An important aspect that has not been considered in those studies is proper outgroup sampling, since a poor choice in this respect can impact phylogenetic reconstruction adversely [17–19]. For example, poor outgroup choice may cause some types of long-branch attraction [20], which may erroneously approximate unrelated branches (e.g., due to convergences). Yet, Zhang et al. [13] used two relatively distant genomes (two *Xanthomonas fuscans* subsp. *aurantifolii*), while Gordon et al. [14] used a single closer strain (*Xanthomonas citri* pv. *bilvae*). Bansal et al. [21] went in the other direction in their sampling scheme, not focusing on XCC pathotype evolution itself (they used a single XCC representative, an A-pathotype genome), but instead aiming at confirming and refining the relationships of a broader set of lineages that they collectively referred to as “*Xanthomonas citri* pathovars” (XCPs), in a phylogenetic analysis using 28 conserved genes. Their genome set had been previously suggested [22] based on *gyrB* sequences. Their phylogeny further confirmed that the XCP genomes were more closely related to XCC than *X. fuscans*, and some of them even closer than *X. citri* pv. *bilvae*. XCP similarities based on ANI and dDDH were also above the cutoffs for considering the genomes included in their work as a single species (values obtained were respectively 98 and 86%, against cutoffs of 95 and 70%). *X. campestris* pv. *durantae* LMG 696 (which infects Verbenaceae plants, a distant family in the asterid clade instead of the more typical rosid parasitism of XCC relatives; Table 1) emerged as the closest relative to the only XCC genome that they used (*X. citri* pv. *citri* LMG 9322); Bansal et al. referred to *X. campestris* pv. *durantae* as a “clonal variant” of *X. citri* pv. *citri* LMG 9322 (based on their comparative genomic analyses), even though it was not clear from their reported phylogeny whether *X. campestris* pv. *durantae* is sister to XCC or clustered within it.

**Table 1:** The 95 genomes validated after selection based on PCA (see text). Column ‘Status’ states whether genome is complete or gives number of contigs if not

Strain	Isolation	Host Taxonomy	Lineage	Location	Reference	Source	Accession/Project	Status*
bilvae_NCPPB3213_India	1980	Rosids:Sapindales:Rutaceae	outgroup	India	[14]	NCBI	CDHI01	60
glycines_CFBP2526_Sudan	1956	Rosids:Fabales:Fabaceae	outgroup	Sudan	[23]	NCBI	AUWO01	complete
glycines_CFBP7119_Brazil	1981	Rosids:Fabales:Fabaceae	outgroup	Brazil	[23]	NCBI	NZ_CM002264.1	complete
malv_X20_Burkina	?	Rosids:Malvales:Malvaceae	outgroup	Burkina Faso	[24]	NCBI	NZ_CM002029.1	complete
mang_LG81-27_Reunion	2009	Rosids:Sapindales:Anacardiaceae	outgroup	Reunion	[25]	NCBI	PEBZ01	6
P._cissicola_LMG21719	1974	Rosids:Vitales:Vitaceae	outgroup	Japan	[21]	NCBI	LOJT01	313
X._axon._bauhiniae_LMG548	1961	Rosids:Fabales:Fabaceae	outgroup	India	[21]	NCBI	LOKR	192
X._axon._cajani_LMG558	1950	Rosids:Fabales:Fabaceae	outgroup	India	[21]	NCBI	LOKQ01	312
X._axon._clitoriae_LMG9045	1974	Rosids:Fabales:Fabaceae	outgroup	India	[21]	NCBI	LOKA01	91
X._axon._khayae_LMG753	1957	Rosids:Sapindales:Meliaceae	outgroup	Sudan	[21]	NCBI	LOKN01	354
X._axon._martyniicola_LMG9049	1958	Asterids:Lamiales:Martyniaceae	outgroup	India	[21]	NCBI	LOJX01	76
X._axon._melhusii_LMG9050	1961	Asterids:Lamiales:Lamiaceae	outgroup	India	[21]	NCBI	LOJW01	101
X._axon._punicae_LMG_859	1959	Rosids:Myrtales:Lythraceae	outgroup	India	[21]	NCBI	CAGJ01	217
X._camp._azadirachtae_LMG543	1971	Rosids:Sapindales:Meliaceae	outgroup	India	[21]	NCBI	LOKS01	236
X._camp._centellae_LMG9044	1979	Asterids:Apiales:Apiaceae	outgroup	India	[21]	NCBI	LOJR01	315
X._camp._durantae_LMG696	1956	Asterids:Lamiales:Verbenaceae	outgroup	India	[21]	NCBI	LOKP01	187
X._camp._leeana_LMG9048	1967	Rosids:Vitales:Vitaceae	outgroup	India	[21]	NCBI	LOJY01	92
X._camp._thespesiae_LMG9057	1978	Rosids:Malvales:Malvaceae	outgroup	India	[21]	NCBI	LOJU01	93
X._camp._viticola	1972	Rosids:Vitales:Vitaceae	outgroup	India	[21]	NCBI	CBZT01	50
X._camp._vitiscarnosae_LMG939	1962	Rosids:Vitales:Vitaceae	outgroup	India	[21]	NCBI	LOKI01	105
X._camp._vitistrifoliae_LMG940	1961	Rosids:Vitales:Vitaceae	outgroup	India	[21]	NCBI	LOKH01	184
X._camp._vitiswoodrowii_LMG954	1961	Rosids:Vitales:Vitaceae	outgroup	India	[21]	NCBI	LOKG01	102
Xc_03-1638-1-1_Argentina_A	2003	Rosids:Sapindales:Rutaceae	A	Argentina	[26]	NCBI	GCA_002952295.1	complete
Xc_306_Brazil_A	1997	Rosids:Sapindales:Rutaceae	A	Brazil	[9]	NCBI	NC_003919.1	complete
Xc_5208_USA_A	2002	Rosids:Sapindales:Rutaceae	A	USA	[13]	NW	NZ_CP009028.1	complete
Xc_AS270_Saudi_Arabia_As	1988	Rosids:Sapindales:Rutaceae	A*	Saudi Arabia	[13]	NW	GCA_000950845.1	29
Xc_AS8_Saudi_Arabia_As	?	Rosids:Sapindales:Rutaceae	A*	Saudi Arabia	[13]	NW	GCA_000950875.1	32
Xc_AS9_Saudi_Arabia_As	?	Rosids:Sapindales:Rutaceae	A*	Saudi Arabia	[13]	NW	GCA_000950855.1	31
Xc_Aw12879_USA_Aw	2000	Rosids:Sapindales:Rutaceae	Aw	USA	[27]	NCBI	NC_020815.1	complete
Xc_AW13_USA_Aw	2003	Rosids:Sapindales:Rutaceae	Aw	USA	[13]	NW	NZ_CP009031.1	complete
Xc_AW14_USA_Aw	2005	Rosids:Sapindales:Rutaceae	Aw	USA	[13]	NW	NZ_CP009034.1	complete
Xc_AW15_USA_Aw	2005	Rosids:Sapindales:Rutaceae	Aw	USA	[13]	NW	NZ_CP009037.1	complete
Xc_AW16_USA_Aw	2005	Rosids:Sapindales:Rutaceae	Aw	USA	[13]	NW	NZ_CP009040.1	complete
Xc_BL18_USA_A	2011	Rosids:Sapindales:Rutaceae	A	USA	[13]	NW	NZ_CP009025.1	complete
Xc_C40_Reunion_A	1988	Rosids:Sapindales:Rutaceae	A	Reunion	[14]	Pruvost	CCWX01	complete
Xc_CFBP2852_India_A	?	Rosids:Sapindales:Rutaceae	A	India	[14]	Pruvost	CCWI01	57
Xc_CFBP2911_Pakistan_As	1984	Rosids:Sapindales:Rutaceae	A*	Pakistan	[14]	Pruvost	CCWD01	87
Xc_FB19_USA_A	2011	Rosids:Sapindales:Rutaceae	A	USA	[13]	NW	NZ_CP009022.1	complete
Xc_FDC1083_Brazil_A	1980	Rosids:Sapindales:Rutaceae	A	Brazil	[14]	Pruvost	CCVZ01	42
Xc_FDC1662_Brazil_A	2011	Rosids:Sapindales:Rutaceae	A	Brazil	This study	BIGA	LAUN00000000	85
Xc_FDC1682_Oman_As	1986	Rosids:Sapindales:Rutaceae	A*	Oman	This study	BIGA	LAUG00000000	168
Xc_FDC217_Brazil_A	2003	Rosids:Sapindales:Rutaceae	A	Brazil	[14]	Pruvost	CCWY01	41
Xc_FDC628_Brazil_A	2001	Rosids:Sapindales:Rutaceae	A	Brazil	This study	BIGA	LAUE00000000	101
Xc_FDC636_Brazil_A	1996	Rosids:Sapindales:Rutaceae	A	Brazil	This study	BIGA	LAUQ00000000	127
Xc_FDC654_Brazil_A	1999	Rosids:Sapindales:Rutaceae	A	Brazil	This study	BIGA	LAUF00000000	114
Xc_FDC828_Brazil_A	1997	Rosids:Sapindales:Rutaceae	A	Brazil	This study	BIGA	LAUP00000000	121
Xc_gd2_China_A	2011	Rosids:Sapindales:Rutaceae	A	China	[13]	NW	NZ_CP009019.1	complete
Xc_gd3_China_A	2011	Rosids:Sapindales:Rutaceae	A	China	[13]	NW	NZ_CP009016.1	complete
Xc_JF90-2_Oman_As	1986	Rosids:Sapindales:Rutaceae	A*	Oman	[14]	Pruvost	CCWA01	85
Xc_JF90-8_Oman_Aw	2002	Rosids:Sapindales:Rutaceae	Aw	Oman	[14]	Pruvost	CCWB01	30
Xc_JJ10-1_Mauritius_A	1985	Rosids:Sapindales:Rutaceae	A	Mauritius	[14]	Pruvost	CDDV01	258
Xc_JJ238-10_Maldives_A	1987	Rosids:Sapindales:Rutaceae	A	Maldives	[14]	Pruvost	CCWC01	56
Xc_JJ238-24_Thailand_As	1989	Rosids:Sapindales:Rutaceae	A*	Thailand	[14]	Pruvost	CCVX01	52
Xc_JK2-10_Saudi_Arabia_As	1988	Rosids:Sapindales:Rutaceae	A*	Saudi Arabia	[14]	NCBI	CCWV01	complete
Xc_JK4-1_China_A	1985	Rosids:Sapindales:Rutaceae	A	China	[14]	Pruvost	CDMR01	320
Xc_JM35-2_Saudi_Arabia_As	1992	Rosids:Sapindales:Rutaceae	A*	Saudi Arabia	[14]	Pruvost	CDMS01	339
Xc_JS581_Iran_As	1997	Rosids:Sapindales:Rutaceae	A*	Iran	[14]	Pruvost	CDAW01	358
Xc_JS584_Iran_As	1997	Rosids:Sapindales:Rutaceae	A*	Iran	[14]	Pruvost	CCWF01	61
Xc_JW160-1_Bangladesh_A	2000	Rosids:Sapindales:Rutaceae	A	Bangladesh	[14]	Pruvost	CCWH01	88
Xc_jx4_China_A	2011	Rosids:Sapindales:Rutaceae	A	China	[13]	NW	NZ_CP009013.1	complete
Xc_jx5_China_A	2011	Rosids:Sapindales:Rutaceae	A	China	[13]	NW	NZ_CP009010.1	complete
Xc_jx-6_China_A	2014	Rosids:Sapindales:Rutaceae	A	China	Chen et al. (unpublished)	NCBI	NZ_CP011827.2	complete
Xc_LB100-1_Seychelles_A	2005	Rosids:Sapindales:Rutaceae	A	Seychelles	[14]	Pruvost	CDAV01	299
Xc_LC80_Mali_A	2006	Rosids:Sapindales:Rutaceae	A	Mali	[14]	Pruvost	CCWJ01	51
Xc_LD71a_Cambodia_As	2007	Rosids:Sapindales:Rutaceae	A*	Cambodia	[14]	Pruvost	CCWE01	49
Xc_LE20-1_Ethiopia_As	2008	Rosids:Sapindales:Rutaceae	A*	Ethiopia	[14]	Pruvost	CCWK01	41
Xc_LG115_India_Aw	2007	Rosids:Sapindales:Rutaceae	Aw	India	[14]	Pruvost	CDAY01	377
Xc_LG117_Bangladesh_A	2009	Rosids:Sapindales:Rutaceae	A	Bangladesh	[14]	Pruvost	CDAX01	338
Xc_LG98_Bangladesh_A	2006	Rosids:Sapindales:Rutaceae	A	Bangladesh	[14]	Pruvost	CDBA01	323
Xc_LH201_Reunion_A	2010	Rosids:Sapindales:Rutaceae	A	Reunion	[26]	NCBI	GCA_001922105.1	complete
Xc_LH276_Reunion_A	2010	Rosids:Sapindales:Rutaceae	A	Reunion	[26]	NCBI	GCA_001922065.1	complete
Xc_LH37-1_Senegal_A2	2010	Rosids:Sapindales:Rutaceae	A	Senegal	[14]	Pruvost	CDAS01	417
Xc_LJ207-7_Reunion_A	2012	Rosids:Sapindales:Rutaceae	A	Reunion	[26]	NCBI	GCA_001922085.1	complete
Xc_LL074-4_Martinique_A	2014	Rosids:Sapindales:Rutaceae	A	Martinique	[26]	NCBI	GCA_001922045.1	complete
Xc_LM180_Argentina_A	2003	Rosids:Sapindales:Rutaceae	A	Argentina	[28]	NCBI	GCA_001939985.1	complete
Xc_LM199_Argentina_A	2015	Rosids:Sapindales:Rutaceae	A	Argentina	[28]	NCBI	GCA_001939965.1	complete
Xc_LMG9322_USA_A	1986	Rosids:Sapindales:Rutaceae	A	USA	[14]	Pruvost	CCVY01	46
Xc_mf20_USA_A	2011	Rosids:Sapindales:Rutaceae	A	USA	[13]	NW	NZ_CP009007.1	complete
Xc_MN10_USA_A	2005	Rosids:Sapindales:Rutaceae	A	USA	[13]	NW	NZ_CP009004.1	complete
Xc_MN11_USA_A	?	Rosids:Sapindales:Rutaceae	A	USA	[13]	NW	NZ_CP009001.1	complete
Xc_MN12_USA_A	1997	Rosids:Sapindales:Rutaceae	A	USA	[13]	NW	NZ_CP008998.1	complete
Xc_NCPPB3562_India_A2	1988	Rosids:Sapindales:Rutaceae	A	India	[14]	Pruvost	CCXZ01	98
Xc_NCPPB3607_India_As	1988	Rosids:Sapindales:Rutaceae	A*	India	[14]	Pruvost	CDAT01	432
Xc_NCPPB3608_India_Aw	1988	Rosids:Sapindales:Rutaceae	Aw	India	[14]	Pruvost	CCWG01	55
Xc_NCPPB3612_India_A2	1988	Rosids:Sapindales:Rutaceae	A	India	[14]	Pruvost	CDAQ01	426
Xc_NIGEB-386_Iran_As	2009	Rosids:Sapindales:Rutaceae	A*	Iran	[29]	NCBI	JRON01	183
Xc_NIGEB-88_Iran_As	2009	Rosids:Sapindales:Rutaceae	A*	Iran	[30]	NCBI	LJGA01	18
Xc_NT17_USA_A	2011	Rosids:Sapindales:Rutaceae	A	USA	[13]	NW	NZ_CP008995.1	complete
Xc_TX160042_USA_Aw	2015	Rosids:Sapindales:Rutaceae	Aw	USA	[31]	NCBI	GCA_002139975.1	complete
Xc_TX160149_USA_Aw	2015	Rosids:Sapindales:Rutaceae	Aw	USA	[31]	NCBI	GCA_002139975.1	complete
Xc_TX160197_USA_Aw	2015	Rosids:Sapindales:Rutaceae	Aw	USA	[31]	NCBI	TX160197	complete
Xc_UI6_China_A	2011	Rosids:Sapindales:Rutaceae	A	China	[13]	NW	NZ_CP008992.1	complete
Xc_UI7_China_A	2011	Rosids:Sapindales:Rutaceae	A	China	[13]	NW	NZ_CP008989.1	complete
Xc_X2003-3218_USA_Aw	2003	Rosids:Sapindales:Rutaceae	Aw	USA	[14]	Pruvost	CCWL01	52
Xc_Xac29-1_China_A	?	Rosids:Sapindales:Rutaceae	A	China	NCBI	NCBI	GCA_000348585.1	complete

Regarding a broader evolutionary perspective, the tempo and mode of XCC evolution has been examined in previous work, but using only a few genes and/or based on discursive biogeographic assertions. Mhedbi-Hajri et al. [32] showed that the ancestor to the larger *X. axonopodis* group (embracing XCPs, hence also XCC, as one of the clades within its descendants) originated at most ~ 25,000 years ago (ya) using a coalescent approach, based on a set of seven housekeeping genes. Biogeographically, the proposition of XCC having originated in Southern China, Indonesia, or India has been advocated [4–6, 32], but until now no area reconstruction appraisal has been carried to test this hypothesis. Moreover, the ancestral host of XCC, which is another important evolutionary information that may shed light on important biological questions, has not been estimated so far.

One important aspect in comparative genomics is the set of genes associated with evolution of different lineages, so that their biological importance through (relative or absolute) time across clades can be inferred. Such an extensive effort of cataloging and discussing gene presence/absence across A, A^w^, and A* genomes can be found in Zhang et al. [13], Gordon et al. [14], and Bansal et al. [21], who found that important virulence/pathogenicity-associated genes belonging in the category of effectors, secretion systems, lipopolysacharides, and other functional groups are differentially associated across pathotypes (or pathotype clades). Other XCC genes inducing pathogenicity in plants continue to be found, mostly tested biochemically in reduced genome sets of the A pathotype alone [33–38]. Furthermore, because there are clear differences in host range and virulence/pathogenicity patterns across the three pathotypes (as mentioned above) but only a handful of genes associated with A* and A^w^ phenotypes have been found so far [13, 14], it is important to expand the search for pathotype-associated suspected genes.

Given the presence of well-supported yet contrasting resolutions of pathotype relationships in the phylogenomic studies of Zhang et al. [13] and Gordon et al. [14], together with the availability of more genomes from the outgroup studied by Bansal et al. [21], we aimed at a more inclusive phylogenomic dataset in terms of both ingroup (XCC) and outgroup (remaining XCP), also including five new A and one new A* genomes sequenced by our group. Besides minimizing artifacts such as some types of long-branch attraction, this inclusive analysis allows finer-grained analyses of presence/absence of genes, biogeographic patterns, and divergence dating estimates due to an increased number of nodes along the phylogeny, making evolutionary transitions detectable at a finer scale. More specifically, our analyses considered different sources of phylogenetic and molecular dating bias (method, dataset, and effect of recombination) that may be impacting the resolution of pathotype relationships and inference of divergence times. At the same time, we inferred the ancestral XCC host, where it originated and when, and whether dispersion or vicariance was the most dominant force in the evolution of XCC. We also assessed 120 pathogenicity-related genes that could have contributed to the evolution of XCC lineages: 63 effectors from the Xanthomonas.org database; and 57 genes with virulence effects whose presence or absence across XCP had not been systematically verified [33–38].

## Results

PCA analyses were helpful at selecting our genome dataset (Additional file 2: Figure S1). The 95 validated genomes (Table 1) were found to contain 1785 unicopy homologous genes, which were multiply-aligned with posterior curation in Aliview [39]. A total of 297 core-LCBs with at least 5000 bp were found by ProgressiveMauve [40], and only five blocks among these lacked significant recombination. The numeric matrix of stretches of indels obtained from SeqState [41] (using modified complex coding) followed by binary recoding had 1247 characters.

The core-genome saturation plots showed conformity to a straight line (Additional file 3: Figure S2), suggesting lack of conspicuous saturation within our data. Furthermore, the chi-squared compositional test in IQTree [42] revealed that no genomes (including those in the outgroup) presented deviant base frequencies. These results suggest that a homogeneous and reversible process of evolution is a reasonable assumption across the genomes studied, thus validating model choice among reversible likelihood models included in IQTree.

**Fig. 1:**
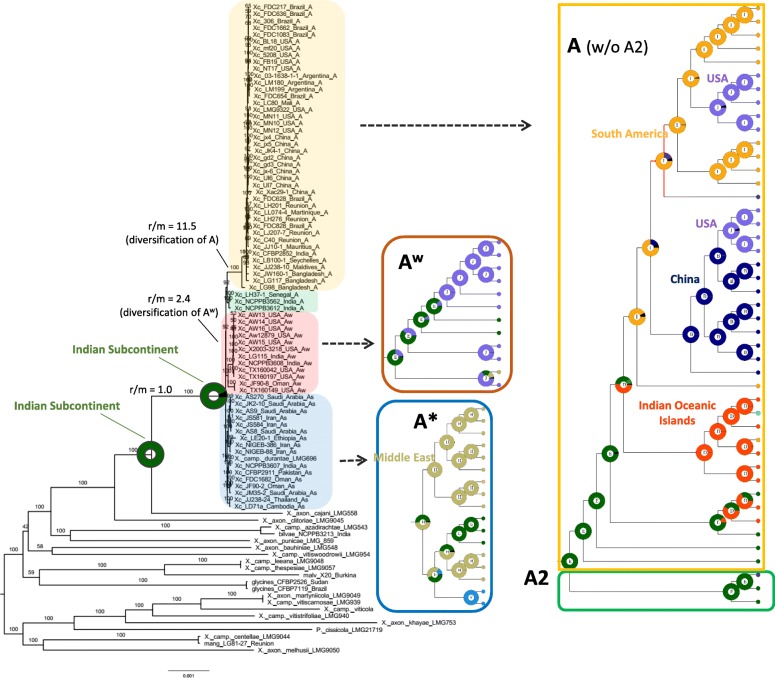
ML tree (model: GTR + I + R2, where “R” means free rate model) based on concatenation of the unicopy data set (1785 genes), with clades zoomed to the right. Ancestral area reconstruction at each node is presented (highly probable ancestral states for nodes discussed in the main text are highlighted, with text color matching state color). “r/m” values correspond to the relative probabilities of a site being altered due to recombination relative to mutation (i.e., r/m = 1.0 means a random site is equaly probable to have suffered recombination or mutation), as obtained in ClonalFrameML

**Fig. 2:**
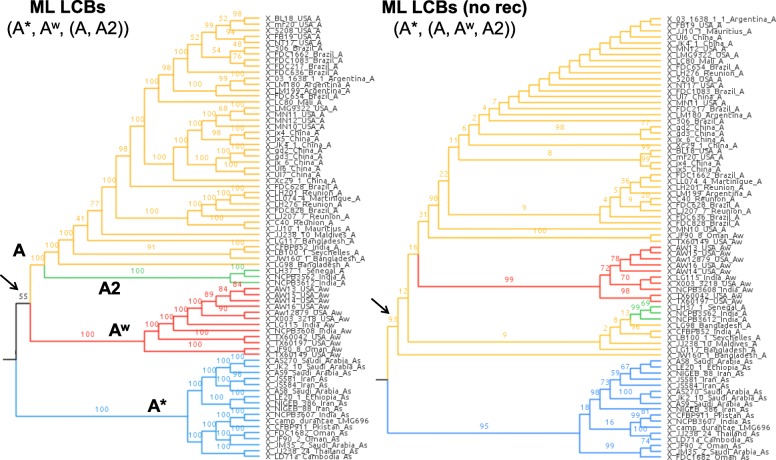
LCB-based ML trees (branches not proportional to actual lengths), either keeping all sites (left), or after removing LCBs with significant signs of recombination (right). Only ingroup is shown. Arrows point to node support associated with the smallest clade containing both A (including A2) and A^w^

**Fig. 3:**
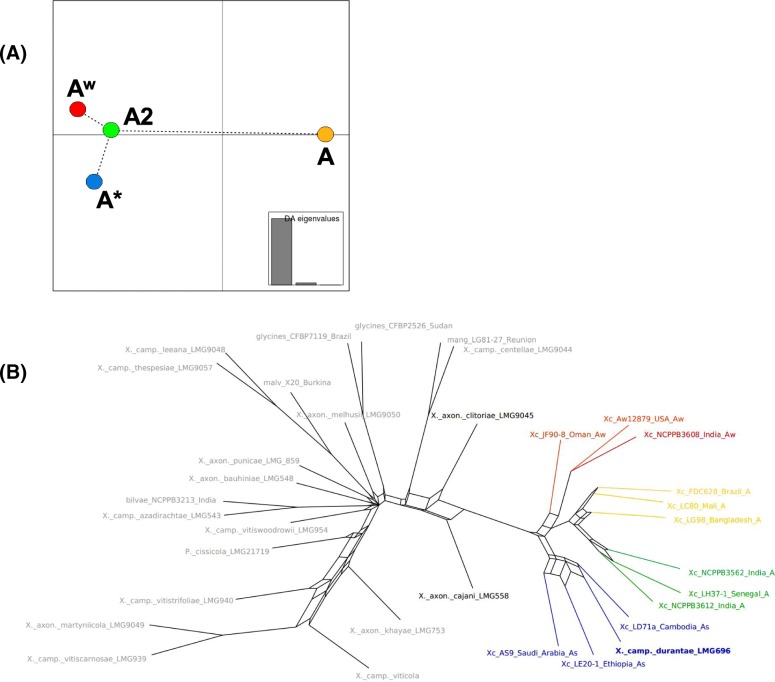
Inference of populations and distances between them according to different analyses. **a** Centroids of populations according to DAPC analysis; **b** Consensus network based on splits present in at least 0.05 of the 161 LCB gene trees for the 34-taxa set

[A + A^w^] was highly supported in the ML unicopy tree (Fig. 1), though also present in other phylogenomic analyses with moderate support (i.e., 50–95%) (Table 2; Additional file 4: Figure S3): ML of LCBs (55%), ML of LCBs without recombination (93%, though A^w^ was monophyletic within a paraphyletic A; Fig. 2), and the LCB species tree method (84%) (Table 2; Additional file 4: Figure S3); the consensus network (no support available; Fig. 3b) and ML indels (46%) further indicated [A + A^w^] as well. On the other hand, the unicopy species tree detected [A* + A^w^] with 100% support, MP unicopy found a [A^w^ + A2] clade (100%) sister to A* (100%) (Table 2; Additional file 4: Figure S3), and the DAPC analysis [43] (Fig. 3a) indicated a closer proximity of A^w^, A*, and A2. This latter clade, which we call A2 (composed of citri LH37_1_Senegal_A, citri_NCPBB3562_India_A, and citri_NCPPB_3612_India_A) had not been named before, although it could be observed in the tree obtained by Gordon et al. [14], and is a clade similar but not identical to the clade DAPC2 described by Pruvost et al. [16]. Clade A2 changed its position across some analyses here as seen above; moreover, no phenotypical differences of its members with respect to the other A strains are known to us. *X. campestris* pv. *durantae*, which was initially considered a close member of the outgroup given a previous phylogeny including a single XCC [21], actually emerged within the A* clade (Figs. 1 and 3b), with ANI distances revealing its closer proximity to an A^w^ genome (TX160149) as well as to other A* genomes. *X. axon. Cajani LMG 558* (*X. cajani*) and *X. axon. Clitoriae LMG 9045* (*X. clitoriae*) were identified as the immediate ancestors of XCC, similarly to the result obtained by Bansal et al. [21].

**Table 2:** Summary of inferred ingroup clades for each phylogenetic method employed. Branch support is shown considering a threshold of 95%

Phylogenetic analysis	Resolution
ML Unicopy	(A*, (A^w^, (A, A2)))
ML LCBs	(A*, A^w^, (A, A2))
ML LCBs (no rec)	(A*, (A, A^w^, A2))
Species Tree LCBs	(A*, A^w^, (A, A2))
ML Indels	(A2, A*, A^w^, A)
Species Tree Unicopy	((A*, A^w^), A, A2)
MP Unicopy	(A, (A*, (A^w^, A2)))

Homologous recombination rates estimated by ClonalFrameML [44] were stable across the two replicate runs, so we provide the mean r/m per-branch between them. The branch leading to XCC diversification had r/m = 1.0, and only two branches in the ingroup had r/m ≥ 2.0 (i.e., the probability of sites being altered by recombination being twice as large as the ones impacted by mutation), with the branches subtending A (to the exclusion of A2) and A^w^ with r/m of 11.5 and 2.4, respectively (Fig. 1).

**Fig. 4:**
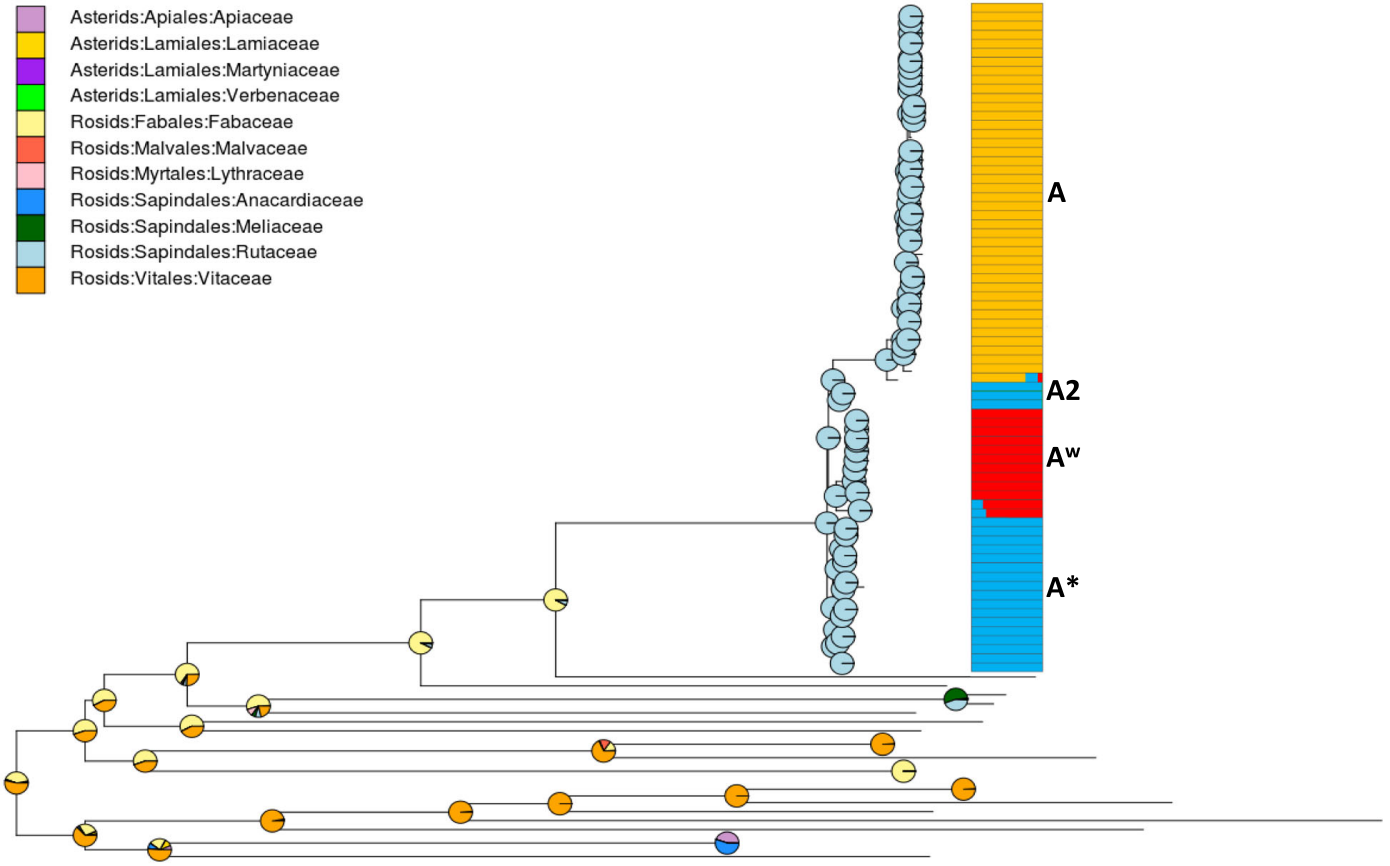
Reconstruction of ancestral hosts at nodes, with pie charts representing the likelihood of inferred states. To the right, best number of inferred populations (k = 3) according to BAPS v6.0, where each genome (individual horizontal bars) has a probability of pertaining to each of the three populations (represented by its proportion of yellow, red, and blue colors)

Area reconstructions at nodes (given the ML unicopy tree) estimated by the Bayesian Binary MCMC method (BBM; modified from [45]) are shown in Fig. 1, suggesting the Indian Subcontinent as the more probable ancestral area from which XCC originated and started to diversify. The complete set of reconstructions across ingroup and outgroup can be found in Additional file 5: Figure S4. Reconstructed hosts at ancient nodes by phytools [46] are shown in Fig. 4. The root state has a large probability of being either Vitaceae or Fabaceae (both Rosids). The largest probability for the two immediate ancestor nodes to the ingroup is Fabaceae as hosts (Fig. 4).

Different runs of the dating analyses converged after at most 100 million (M) generations. The strict clock hypothesis was rejected by treedater ([47]; *p* < 0.05). Root-to-tip regressions in TempEst [48] showed lack of association between tip-dated times (isolation dates) and root-to-tip length, for either XCP (R^2^ = 0.08) or XCC alone (R^2^ = 0.15), revealing tip-dating was uninformative regarding dating purposes. Given these results, we proceeded with molecular dating using the UCLN model in Beast v1.10.4 [49] without incorporating tip-dating.

**Fig. 5:**
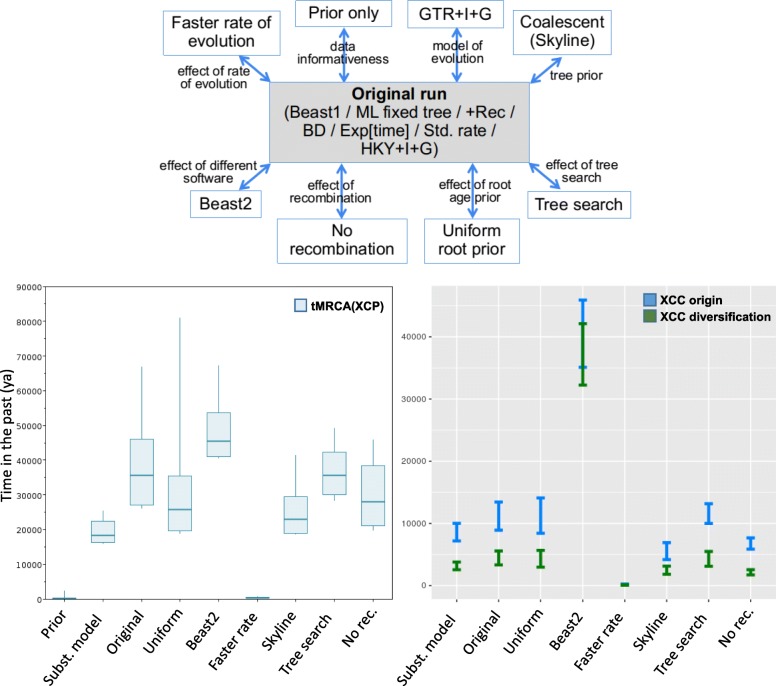
Dating analyses summary. *Top:* the eight tests performed, each changing a parameter. *Bottom left:* tMRCAs of the root (= start of diversification of the XCP group), with box borders corresponding to 95% HPDs. *Bottom right:* times of origin and diversification of XCC (95% HPDs)

Dating runs are further summarized in Fig. 5, with times and AICM values (a posterior simulation analog of the Akaike’s information criterion; [50]) obtained from the posterior summarized in Table 3 (in order of decreasing fit). The prior distribution using the exponential without data for the root did not overlap the regular run with data, so the 34-taxa LCB dataset was deemed informative for divergence dating. The more complex GTR + I + G model (instead of HKY + I + G) had a substantially higher fit than other scenarios tested, while performing tree search concomitantly with dating had the worst fit (Table 3). The prior run and the analysis without recombining regions (“No rec.”) could not be compared to the others using AICM, because alignment data was either absent (prior) or was different (no recombination).

**Table 3:** Dating models implemented, respective 95% HPDs, and their relative fit (by AICM and ΔAICM). Models are ranked decreasingly from top to bottom (lower AICM values corresponding to better fit)

BEAST run	tMRCA (XCP)	Origin (XCC)	Diversification (XCC)	AICM	ΔAICM	ΔAICM (compared to Original)
Substitution model	[16206.85, 22464.93]	[7194.44, 10025.40]	[2559.78, 3791.96]	4416757,966	-	-
Original	[27036.54, 46090.070]	[8916.42, 13441.22]	[3335.72, 5568.49]	4417749,144	-991,18	-
Uniform	[19602.58, 35489.83]	[8424.34, 14096.51]	[2972.82, 5663.46]	4417754,423	-996,46	5,28
Original (BEAST2)	[41056.62, 53663.97]	[35100.86, 45893.22]	[32245.31, 42118.59]	4417757,026	-999,06	7,88
Faster rate	[242.83, 447.08]	[141.46, 259.28]	[69.37, 130.39]	4417766,797	-1008,83	17,65
Skyline	[18862.65, 29544.91]	[4195.23, 6915.09]	[1820.51, 3132.74]	4417851,234	-1093,27	102,09
Tree_search	[29956.02, 42231.86]	[10006.82, 13174.56]	[3111.25, 5492.1]	4418106,267	-1348,30	357,12
No_Rec	[21135.18, 38328.66]	[5865.59, 7673.84]	[1730.30, 2565.84]	-	-	-
*** Conservative time spans ***						
tMRCA (XCP) =	[16206.85, 46090.070]					
Origin (XCC) =	[5865.59, 14096.51]					
Diversification (XCC) =	[1730.30, 5663.46]					

We carried out a presence/absence analysis based on tBLASTn searches of 120 pathogenicity-associated genes (Additional file 1: Table S2), 59 of which were universally present, 17 were absent from all genomes, and the remaining 44 showed some level of polymorphism. Based on variable presence in the A, A^w^, and A* pathotype genomes, we highlight the following results: *xopJ5* is absent in all XCC strains but present in most outgroup genomes; *xopAG* is present only in A^w^ among ingroup strains; *xopAF2* is lacking in most A strains, but present in all A* and A^w^ strains and two of three A2, among ingroup strains; the uncharacterized gene XAC1496 is absent from A* strains among those in the ingroup; *xopT* was identified in nine A* strains and in only one A^w^ strain, besides a few outgroup genomes; and *xopC1* was identified in 10 A* strains regarding the ingroup.

## Discussion

The phylogenetic pattern most common throughout the analyses, [A + A^w^], though always with low support except for ML unicopy agreed with a previous work [14], while one of the phylogenies (species tree unicopy) agreed upon [A* + A^w^ + A2] (Additional file 4: Figure S3), more in line with Zhang et al. [13]. Gordon et al. [14], which used a more extensive dataset with regions of recombination removed, argued that the result by Zhang et al. could be explained by the latter authors not having removed recombinant regions. However, Zhang et al. [13] used ClonalFrame [44] in their paper, a method that corrects for the effect of recombination, and yet they obtained the same tree as with ML with all genes. Recombination being disregarded as a possible bias in their case, there might be unnoticed biases in Zhang et al. [13] such as inclusion of an overly distant outgroup (*X. fuscans*), or the fact that they used only 23 genomes in total (including the outgroup). On the other hand, in our case, accounting for the impact of recombination increased our confidence in the [A + A^w^] relationship: by excluding LCBs with significant signs of recombination, the branch support for this clade increased noticeably, from 55% (all LCBs) to 93% support (including only LCBs without recombination) (Fig. 2; Additional file 4: Figure S3).

The impact of genetic similarity between non-sister XCC clades can be observed in BAPS [51], DAPC, and network analyses (Figs. 3 and 4). In the case of BAPS, the A2 individuals were shown to be highly similar to A* (instead of being more similar to A), to a point of being considered a single population altogether (Fig. 4). This is inline with the DAPC analysis, where genomes of the A2 group were placed in an intermediate position between A and the other pathotypes (Fig. 3a), apparently making more likely the hypothesis of shared polymorphisms with A* strains (as suggested by Fig. 4); the consensus network further indicates that some paths leading to A2 strains (and also A) could have arisen from a common ancestor shared with A*, even though the strongest signal in the network is of [A + A2 + A^w^] (Fig. 3b). However, going one step further, A2 bears relatively low r/m values (as well as A*), which may suggest that the shared polymorphisms of alleles in the A2 clade (and A*) are not due extensively to genomic imports after divergence of the pathotypes, but possibly due to other factors such as retention of ancestral polymorphisms, which may have happened if events of successive pathotype divergences happened in a short time within a large ancestral effective population number (N_e_) (which can happen by mutation alone if bacterial populations evolve for sufficient time). In this sense, one likely outcome of successive speciations is the presence of small branch lengths in between them, which is a feature revealed by most phylograms inferred here (Fig. 1; Additional file 4: Figure S3). On the other hand, the reticulations in the branches leading to A and A^w^ genomes (Fig. 3b) can be better explained by high levels of recombination, because r/m for A was 11.5 (a very high value), and for A^w^ was 2.4.

Overall, a likely scenario is XCC lineages diversifying relatively fast from the ancestral population (possibly with relatively large N_e_ across diversifications), with A* and A2 maintaining a significant number of ancestral polymorphisms, whereas A (disregarding the subclade A2) and A^w^ were more impacted by recombination effects by receiving genomic imports after these lineages had already diverged from their last common XCP ancestor. A great amount of reticulation is also found within the outgroup, suggesting pervasive recombination (among other possibilities) was also important for the evolution of the XCP members (Fig. 3b).

Branch levels of r/m can be further compared to values from Vos and Didelot [52] (see their Table 1) based on reanalyses of previously published data sets. Of special note is the r/m of the branch leading to XCC A diversification (11.5), a value larger than another highly recombining Gamma-proteobacterium within the order Pseudomonadales, *Moraxella catarrhalis* (r/m = 10.1), a commensal of the upper respiratory tract in humans. Values can also be compared to two data sets of a genus from a closely related family (Gamma-proteobacteria: Pseudomonadaceae), both including phytopathogens, *Pseudomonas viridiflava* [53] and *P. syringae* [54], with global levels of r/m respectively of 2.0 and 1.5. As mentioned above, different branches within the evolution of XCC lineages have values larger than those (Fig. 1). In a study with species more closely related to XCC (not focused on sampling of pathotypes), Bansal et al. [21] found an overall r/m = 2.24, a relatively high value compared to the *Pseudomonas* datasets mentioned above, but also within the range of some values observed in Fig. 1. Corroborating such findings, a study [55] inferred that 10% of the core genome of a dataset comprising different *Xanthomonas* species were impacted with homologous recombination. In fact, Mhedbi-Hajri et al. [32], Zhang et al. [13] and Gordon et al. [14] had already observed that the impact of recombination has been quite severe on *X. citri*-like lineages as well. Overall, such results highlight the importance of recombination on the origin and diversification of XCC clades, and on a more general level its importance in related families of pathogenic Gamma-proteobacteria.

**Fig. 6:**
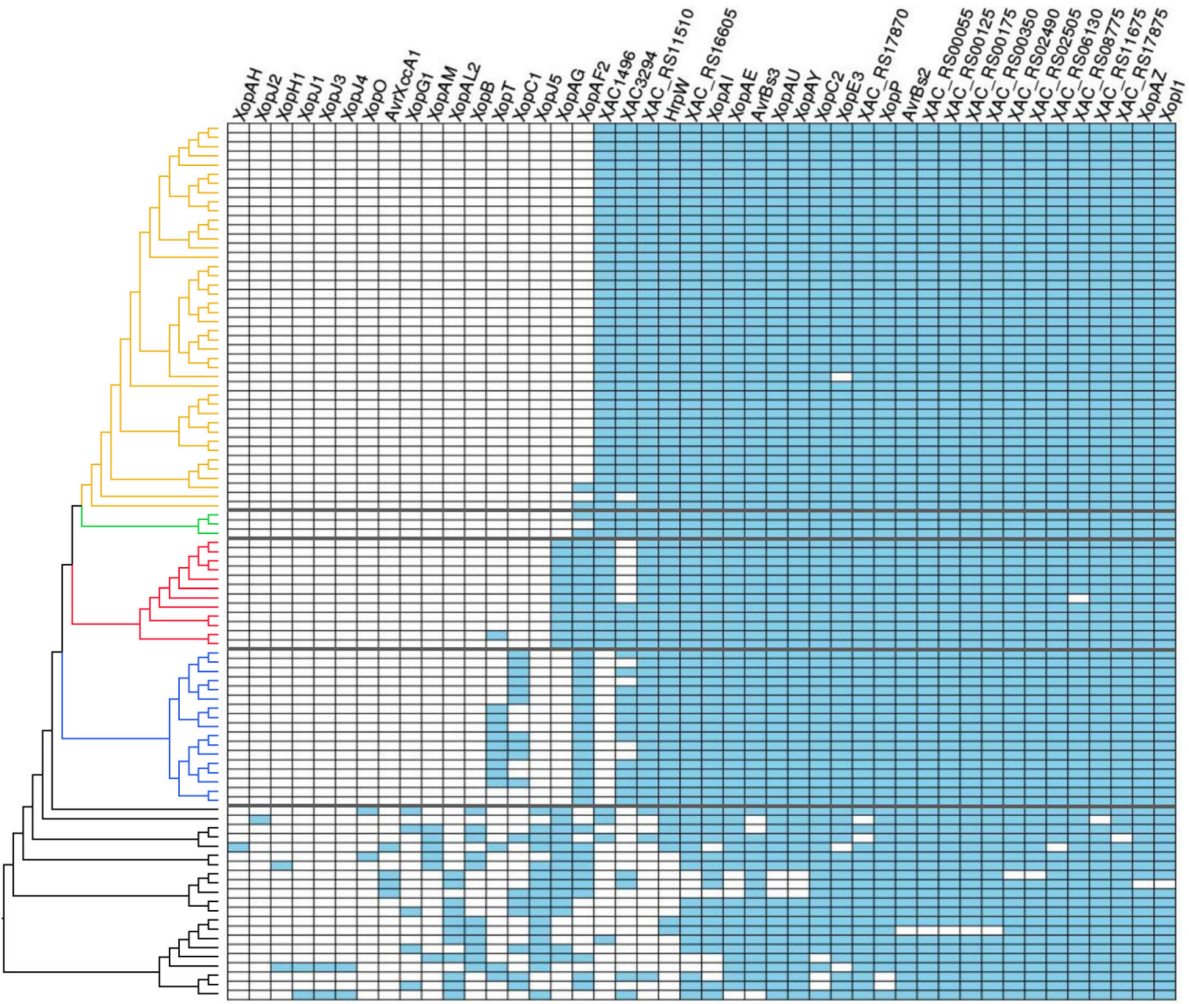
Heatmap of presence/absence of the 44 genes with variable pattern of pathogenicity/virulence (among a larger set of 120 genes), across the 95 genomes (ML tree shown to the left)

An unexpected result was *X. campestris* pv. *durantae* LMG 696 falling inside the A* clade. This strain was paired with *X. citri* pv. citri LMG 9322 in the Bansal et al. [21] phylogenetic analysis (a genome also present in our study, placed in the A clade), even though it infects plants of the Verbenaceae family (within asterids) [56]. We can be reasonably sure that the sequenced genome is a legitimate A* strain, as it displays a pattern similar to other A* genomes for the 44 genes with variable presence/absence (Fig. 6). Bansal et al. [21] reported finding a “large dynamic region” (their term) of 27 kbp in this genome containing genes related to the type IV secretion system, among others. We checked this statement and determined that the region is part of contig 29, which is 42,744 bp long. Approximately 40 kbp of this contig (containing the above region) align with regions in the three sequenced plasmids of *Xanthomonas citri* pv. *citri* strain TX160149, an A^w^ genome (Additional file 6: Figure S5). Furthermore, the region in question is also found in plasmids of *X. campestris* pv. *campestris* strain CN18 (GenBank Biosample SAMN05791239) and *X. campestris* pv. *campestris* strain CN03 (GenBank biosample SAMN02645665), which have as hosts *Brassica* plants. In any case, assuming all published information regarding *X. campestris* pv. *durantae* LMG 696 is correct [56], this suggests that in strains LMG 696, TX160149, CN18, and CN03 transient plasmids may be a factor associated with host range.

Regarding molecular dating, as discussed above (also Additional file 3: Figure S2), there were apparently no large saturation effects on our data, and therefore the effect of underestimating rates on more ancient branches (hence overestimating ancient node times) is apparently minimized. This further suggested the use of a HKY + I + G model throughout most analyses as a reasonable choice, given its speed of convergence of the MCMC chains (data not shown); nevertheless, by using the more complex GTR + I + G, we attained the largest ΔAICM value increase, as well as the most recent HPD times (not considering the test for “faster rates”, see below), suggesting some rate correction was still needed (Table 3). This further reiterates the bias that underparameterized models can inflict on dating estimates [57]. Regarding use of a uniform root prior (instead of exponential), we mention that the original hard upper bound on the root (25,000 ya) showed up in the posterior as a distribution conspicuously stacking to this upper limit, suggesting a larger prior bound was needed, which after trial and error was set to 100,000 ya, correcting the stacking pattern. The uniform prior did not inflict large differences on estimated times (Table 3); furthermore, the AICM values were better than in the remaining tests, so we suggest priors for other parameters could be tried before such a test, in cases of analyses of large bacterial alignments based on a single dating calibration without substantial saturation.

The Beast2 run had worse (higher) AICM value than the above, and furthermore it showed an overlap between divergence times of XCC origin and diversification (Figs. 5; Table 3), a feature that is not present in any of the ML trees computed (Fig. 1; Additional file 4: Figure S3), nor across the Beast v1.10.4 runs (Fig. 5; Table 3). This is so even after we matched prior distributions for all parameters in Beast2 (as many of them change between the two versions), suggesting the reimplementation of the software may be inducing subtle differences (in at least some datasets) that may have not been acknowledged thus far. This odd XCC overlap feature, together with the fact that time ranges were significantly older compared to all other runs (Fig. 5), precluded its time ranges to be included in the final HPDs.

All runs performing worse than the above had significantly worse AICM (Table 3) when compared to the original run (disregarding the GTR + I + G run), after Burnham and Anderson [58], who mention that a ΔAICM value > 10 is sufficient to consider a model unlikely. We therefore disregard dating times returned by those tests as well, though we acknowledge that some of them returned HPDs overlapping the most likely models (Fig. 5; Table 3).

We mention particularly the test for faster rate, in which the upper rate bound of the prior was higher by two orders of magnitude (1e-7 in the original run, to 1e-5 s/s/l/y after [59]); for this dataset, dates were very recent (as the posterior on rates abounded to the faster values), and as mentioned, the AICM value is substantially worse (Fig. 5; Table 3); indeed, the closer relative to *Xanthomonas* in the Duchene et al. dataset [59] is *Pseudomonas aeruginosa*, still quite distant from our target taxa (and yet with rates lower than 1e-6 s/s/l/y). We therefore suggest caution when considering priors (and interpreting posteriors) embracing such high rates.

The coalescent (skyline [60]), a prior supposedly suitable to species-level analyses, performed relatively bad, even though Bansal et al. [21] inferred by ANI and dDDH that all XCPs belong in the same species. Whatever the reason, in terms of date overlaps with other tests, these were comparable to the GTR + I + G run based on a birth-death prior (Fig. 5; Table 3). This is in agreement with a study by Ritchie et al. [61] showing that dates returned by either the birth-death or skyline priors may not strongly affect Bayesian molecular dating estimates.

Tree searching concomitantly with molecular dating had the worst AICM value. This may be due to the clock model and topological search interacting in a non-linear way, biasing times altogether in the process. This raises the question of whether topological search in Beast really aids at estimating divergence times or even at finding the best tree, as originally suggested by the Beast developers [62].

HPDs of XCC origin and divergence were more recent when recombining regions were removed from the dataset, but its model likelihood is not comparable to the other runs. This suggests that comparing dates between recombining vs. non-recombining datasets may be interesting to provide conservative time estimates; we therefore embraced such date estimates in the reported HPDs (Table 3).

In a previous study [32], the authors reconstructed the phylogeny of the more inclusive *X. axonopodis* group using seven housekeeping genes, clarifying the relationship among the six groups proposed earlier [63] (groups 9.1–9.6). XCC clustered within group 9.5 [21], with the time to the most recent common ancestor of this group (tMRCA) being also a conservative upper bound for the tMRCA of XCP (including XCC) regarding comparisons to our dates. The authors found that such an ancestor existed ~ 7900 ya (95% C.I. = 3800–25,800 ya), younger than our root estimate (16,206–46,090 ya) though with considerable range overlap. Such discrepancy may be due to Mhedbi-Hajri et al. [32] having used a contrived set of markers (seven genes) that may lack power when compared to a larger marker set, because the more genes analyzed, the more likely to find markers with different rates that can be informative at estimating divergence times in different temporal scales. Another possible reason is that their taxon sampling was not as inclusive as ours. Finally, they used a coalescent procedure to infer times of evolution, and coalescent approaches could underestimate time divergences if such a model is not very likely - in fact, our coalescent model had significantly worse fit than using a birth-death prior (Table 3); moreover, coalescent approaches for assessment of microbial demography may be misleading even after testing for the best population model (e.g., constant, exponential, skyline, etc.) as it can get biased quite easily depending on the taxonomic inclusion in each lineage [64], which in turn could also bias divergence times.

Altogether, our dating analyses strongly indicate that origin and diversification of XCC occurred after the Last Glacial Maximum (which conservatively started no younger than 19,000 ya [65]), and at a time when deglaciation was on its course in the Northern hemisphere (14,500 ya [65]) facilitating human dispersion and the establishment of plant domestication. Furthermore, times of XCC diversification (1730–5663 ya) coincide with a triangulation of archaeobotanical reports together with critical linguistic analyses based on early Indian Subcontinent and Chinese reports, which indicate that by 2500 ya (and possibly even by 3500 ya) the spread of Citrus cultivars was already taking place in the Middle East and Eastern Asia [66]. The datings we propose for XCC are also much more recent than the hypothesized date of origin of the *Citrus* genus (6 to 8 Mya) [67, 68], suggesting that cross-infection by dispersion was an important trigger for the evolution of pathotypes, instead of host-driven speciation.

The analysis of ancestral hosts indicated with high likelihood that the two immediate ancestral nodes of *X. citri* (leading also to extant *X. a. clitoriae* and *X. a. cajani*) infected Fabaceae, suggesting a host jump from the latter to citrus plants (Rutaceae). XCC can be rapidly dispersed by rainwater, strong winds, and high temperature [69], and also by the agricultural interchanges between citrus-producing countries. All these conditions are met in the Indian Subcontinent, making it a likely source of ongoing spread of new XCC lineages; indeed, most deeper nodes within the phylogeny indicate origin within that region (Additional file 5: Figure S4). We infer that North-American A strains originated from at least two dispersion events, one coming from South America, the other from China (Fig. 1), a pattern that can be better observed due to inclusion of the five newly sequenced Brazilian genomes. Furthermore, we noticed a cluster of samples that apparently spread from recurring Indian Ocean Island ancestors, suggesting fast dispersal between these islands (Fig. 1). In the A^w^ clade, another North-American related reintroduction event emerged from the Indian Subcontinent. In A* strains, a pattern of apparently ongoing middle-eastern recolonizations have been occurring, either unidirectionaly (Fig. 1; A* top clade) or bidirectionaly (Fig. 1; A* bottom clade).

**Fig. 7:**
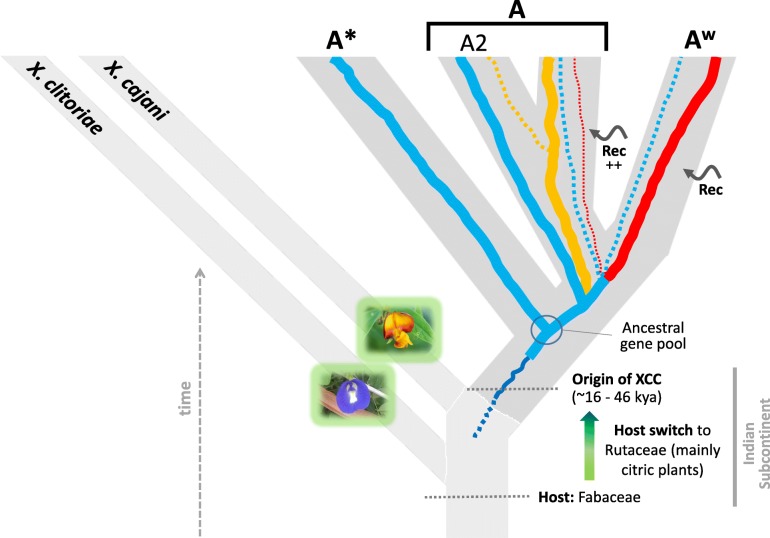
Schematic view of the main results regarding evolution of *X. citri* subsp. *citri*. The lineage originated ~ 16.0–46.0 thousand years ago (kya), with an associated event of host switch from Fabaceae to Rutaceae, within the Indian Subcontinent. A* and A2 likely share a great portion of ancestral polymorphism, whereas A and A^w^ had a larger impact from recombination (“Rec”) on their genetic varibility (especially in A, the generalist pathotype) prior to each respective diversification. Colors of the most common haplotypes in each lineage are the same as in previous figures (except for A2, which due to its high genetic similarity to A* according to BAPS v6.0, is also shown in blue). Dotted lines correspond to minor genetic contributions from given haplotypes (as detected in BAPS v6.0) or inferred from the ML-unicopy phylogeny. Fabaceae images obtained and modified from the Encyclopedia of Life database: “*Clitoria ternatea*” (https://eol.org/pages/47317701; copyright: Vinoth Kumar Rajalingam; license: cc-by-nc-4.0), and “*Cajanus cajan* (L.) Millsp.” (https://eol.org/pages/643268; copyright: Andres Hernandez S.; license: cc-by-nc-sa)

Notwithstanding, we acknowledge that some area reconstructions, as well as the inference of some ancestral hosts, may be incorrect due the effect of unsampled populations, which could interfere with ancestral state estimation. In this sense, it is worth noting that even with the more inclusive outgroup set, there is still a large taxonomic gap between origin of XCC and the start of its diversification, for each dating scenario tested (Table 3; also reconstructed trees in Fig. 1 and Additional file 4: Figure S3 regarding the respective branch), so that their ranges do not even overlap by thousands of years in each such scenario. An example of such a possibly biased biogeographical inference (though not related to the aforementioned branch) is the unlikely dispersal to China coming from South America (Fig. 1), which was probably inferred as such due to a single South American strain hanging alone as the outgroup to a larger clade containing both New World and Old World lineages, therefore “attracting” the South American state to their common ancestral node. A schematic view summarizing our evolutionary inferences is shown in Fig. 7.

The analysis of presence/absence of 120 pathogenicity-associated genes previously screened in XCC A strains revealed interesting patterns. We identified a set of 60 genes present in all XCPs, 18 of them being effectors (*hpaA*, *xopAD, xopAK, xopAP, xopE1, xopE2, xopF1, xopF2, xopK, xopL, xopM, xopN, xopQ, xopR, xopS, xopV, xopX,* and *xopZ1*). A set of six genes showed marked differences across XCC pathotypes, or between ingroup and outgroup. The differential presence across XCC for three of these genes (effectors *xopAF2*, *xopT*, and *xopJ5*) is a novel result. *XopAF2*, related to the avrXv3 of *X. campestris* pv. *vesicatoria,* which elicits resistance response in a specific tomato line [70], is present in all A* and A^w^ strains, in two A2 strains, and absent in the other A strains except for two basal strains. *XopT* [71] is present in nine A* strains and in one A^w^ strain. Bansal et al. [21] indicated that *xopJ5* and *xopC1* were absent from a few XCP genomes; our results are similar, though two subclades of A* (and a separate individual from this clade) bear *XopC1* (Fig. 6), agreeing also with results by [13]. *XopJ5* is the only effector in the 120-gene set that is absent from all XCC genomes. *XopAG*, first reported by Rybak et al. [72], restricts host range and causes hypersensitive response in sweet orange and grapefruit. The result that *xopAG* is restricted to A^w^ is not new, but is mentioned here for completeness. Finally, absence of the uncharacterized gene XAC1496 in A* was observed by Gordon et al. [14]); this gene is associated with a strong chlorosis effect though without visible lesions on host plants, similarly to the effect caused by the highly virulent pthA4 gene of XCC [35]. These results may contribute to future experimental assays that may elucidate the role these genes might play in citrus canker, as well as allowing screening in the hosts of the respective pathotypes for genes associated with resistance.

## Conclusions

Knowing whether it is likely or not for a presently restricted lineage to infect new hosts is highly relevant because such an adaptation could greatly increase the effects caused by the pathogen. It is also interesting to know the timeframe of evolution of known lineages, since this may provide clues for the likelihood of a highly resistant strain emerging in the near future. In this sense, knowing the strength of evolutionary forces such as recombination on a lineage-by-lineage basis may tune this concern more appropriately, because if recombination is more common than expected in specific lineages within a species, more attention can be directed towards them, as they bear an increased risk of outbreaks in case they acquire virulent allelic variants. Moreover, due to the constant arms race between pathogen and host, new genomic targets need to be searched on a regular basis, preferentially with a thorough evolutionary analysis of one or a few genes with major virulence/pathogenic effects in order to infer how susceptible they are to forces such as gains, losses, and horizontal transfers.

With such focal points in mind, we interrogated a vetted dataset of 95 XCC genomes with the largest taxonomic inclusion (ingroup and outgroup) to date. By carrying a thorough phylogenomic investigation (better sampling, use of different genomic regions, use of parametric and non-parametric phylogenetic methods, impact of population structure), we confirmed the presence of an [A + A^w^] clade as observed in a previous study [14]. Important clues obtained here led to the hypothesis that evolution of XCC pathotypes operated by retention of ancestral polymorphisms and recombination, likely blurring part of the phylogenetic signal. Recombination may have been significant in the outgroup taxa too, revealing a complex history involving XCC pathovars. This is in agreement with a previous phylogenomic study of XCC pathotypes [14], which detected different genomic regions involved with recombination, many of them including genes with a role in virulence.

We also conducted thorough molecular dating analyses to test for the impact of different assumptions on origin and diversification times (substitution model, root age prior, rate prior, tree prior, tree search vs. fixed ML tree, including/excluding recombining regions) to infer conservative 95% time intervals, which indicated that the origin of XCC may have occurred after the Last Glacial Maximum.

Having estimated the best tree and divergence times, and further conducting biogeographical analyses, we were able to infer that the XCC ancestor probably made a host-jump from Fabaceae to Rutaceae plants, in the Indian Subcontinent, and with multiple recent dispersals to North America, possibly due to worldwide import/export activities in the Citrus industry.

Taken together, these results provide novel insights into the evolutionary history of XCC as well as a sound phylogenetic foundation for future evolutionary and genomic studies of their pathotypes.

## Methods

### Media and culture conditions of the six new genomes

The six new genomes here presented were sequenced from strains indicated in Additional file 1: Table S1. All strains were stocked both in autoclaved tap water at room temperature and at − 80 °C in NB medium (3 g/L meat extract, 5 g/L peptone) containing 25% glycerol. Each strain was recovered from a − 80 °C stock, streaked on solid NA medium (3 g/L meat extract, 5 g/L peptone and 15 g/L agar) and cultivated for 48 h at 29 °C. For each strain, colonies were inoculated into 10 mL of liquid NB medium in a sterile 50 mL Falcon conical centrifuge tube and incubated at 29 °C in a rotary shaker at 180 rpm for 16 h (final OD600nm ~ 1.0).

### DNA extraction and quantification

A volume of 2 ml of the culture was centrifuged at 16,000 g for 10 min at 4 °C in a refrigerated benchtop microcentrifuge. The supernatant was discarded and the cells pellet was resuspended in 600 μL of Nuclei Lysis Solution supplied by Promega Wizard Genomic DNA purification kit (Promega Corporation, Madison, USA). Total DNA extraction was performed using Promega Wizard Genomic DNA purification kit according to manufacturer instructions. DNA quantity and quality were determined using Nanodrop ND-1000 spectrophotometer (NanoDrop Tech, Wilmington, DE), Qubit 2.0 fluorometer (Invitrogen, Life Technologies, CA, USA) and 0.8% agarose gel electrophoresis. Each extraction yielded at least 5 μg of high-quality genomic DNA.

### Genome sequencing and assembly

The new genomes were sequenced using the Illumina HiScanSQ plataform. An average of ~ 20 M (2 × 100 bp) reads for each genome was generated (Additional file 1: Table S1). The raw reads were trimmed with seqyclean software (https://bitbucket.org/izhbannikov/seqyclean), using minimum phred value of 23, minimum read length of 30 bp, and removing custom Illumina TruSeq adapters. Genome assembly was carried out with SPAdes v3.8.1 [73] with default parameters. Potential plasmid derived scaffolds were identified with plasmidSPAdes v3.8.1 [74].

### Annotation of the genomes

We considered the inclusion of 107 genomes (XCC plus outgroup) available at least as contigs and/or scaffolds available in GenBank as of July 2018. This list included all publicly available XCC genomes, plus the six newly sequenced genomes by our group. We annotated all genomes with DFAST [75] using an augmented database of complete *Xanthomonas citri* (and outgroup) complete genomes. The six genomes that we sequenced were further reannotated with the NCBI Prokaryotic Genome Automatic Annotation Pipeline [76] and submitted to GenBank, with accession numbers given in Table 1.

### Genome validation for phylogenomic analyses

In order to filter out genomes with relatively unwarranted characteristics (that can be obtained from assembly and annotation reports) that could increase the risk of suspicious results substantially, we applied a principal components analysis (PCA) to the 107 genomes including the following features: Total Sequence Length (bp), Number of Sequences, Longest Sequence (bp), N50 (bp), Gap Ratio (%), GC content (%), Number of CDSs, Average Protein Length, Coding Ratio (%), Number of rRNAs, Number of tRNAs, and Number of CRISPRs. After the PCA was completed, we: (1) detected the largest separation between points according to the first PC, treating genomes on each side as two different groups; and (2) removed from downstream analyses genomes of the (so defined) group having worse-behaviored genomes according to one or more of the 12 features above (e.g., having more gaps; or larger N50). The group with lower values in the PC1 axis (Additional file 2: Figure S1) always had smaller Average Protein Length and Coding Ratio, and at the same time their Gap Ratio was higher, these three characteristics being indicative of relatively poorer sequencing and/or assembly. This group was formed by a total of 12 genomes that were further discarded, resulting in a list containing 45 A, 16 A*, 12 A^w^ genomes, plus 22 related genomes supposedly from the outgroup, for a total of 95 included genomes (Table 1).

### Unicopy gene families

The protein-coding genes of the 95 genomes were input into Get_Homologues [77] for gene family clustering using the OMCL option (‘-M’). This setup first produces all-vs-all BLASTp [78] comparisons between predicted protein products, and then runs OrthoMCL [79]. We used thresholds of 80% for both coverage and identity. After the homologous gene families were clustered, the compare_clusters.pl script (within Get_Homologues) was run to obtain the set of genes of single copy present in all 95 genomes, which in principle can be considered to be enriched with vertical phylogenetic signal [80]. Multiple sequence alignment (MSA) of each core-genome single copy gene family was performed by Muscle [81]. Each MSA was then manually checked in Aliview [39], and whenever local regions were suspected of misalignment we used the software’s “realign selected block” option (again using Muscle, within Aliview), to minimize impact of alignment biases in downstream analyses. The vetted alignments were further concatenated into a supermatrix by FasConCAT v1.0 [82] for phylogenomic analyses. This set is referred in the text as *the unicopy dataset*.

### Locally Colinear blocks (LCBs)

We also employed core-LCBs for phylogenomic analyses due to their multiple alignments being independent of annotation biases (if a gene is missing from the reference genomes then it may be left out in other genomes too, and contrarily a wrongly inferred gene annotation can also be perpetuated across genomes), while at the same time allowing larger segments (as 5000 pb is much larger than the average bacterial gene length of ~ 1000 bp) therefore bringing more power to some of the analyses (such as inference of gene trees; see below). We identified LCBs ≥5000 bp using ProgressiveMauve [40], which automatically aligned each of them. The core-LCBs (from here on, simply LCBs) were obtained from this larger LCB set by running stripSubsetLCBs (http://darlinglab.org/mauve/snapshots/2015/2015-01-09/linux-x64/).

### Phylogenomics and network estimation

Saturation plots were obtained with genetic distances calculated using the F84 substitution model [83] in DAMBE [84], to assess possible saturation effects that could compromise phylogenetic estimation and dating analyses [85].

Phylogenetic inferences of the unicopy dataset were done by Maximum Likelihood (ML) and Maximum Parsimony (MP), to test for methodological biases. ML analyses and UFBoot branch support [86] were obtained in IQTree [42]. During substitution model assessment each model was tested for rate variation across sites with a discretized gamma distribution of rates and/or proportion of invariant sites, and alternatively with 2 to 5 rate-across-sites mixture matrices. MP with the unicopy set was run in MPBoot [87], with branch support obtained by 1000 UFBoot pseudoreplicates.

LCBs were analyzed either separated (as “gene trees” for a consensus network), concatenated, or analyzed under a species tree paradigm. ML trees were inferred by either using the whole LCBs concatenated, or else by removing blocks with signs of recombination (see below for details). A consensus network based on LCB gene trees was employed in SplitsTree4 [88] with a threshold of 0.05 (i.e., with tree splits appearing in at least 5% of the gene trees) to assess qualitatively the amount of reticulations leading to pathotype lineages (because some reticulations may be indicative of HGT). Only three genomes from each pathotype were maintained for this analysis (plus all putative outgroup genomes, totaling 34 genomes) to avoid excess of detection of recombination events in terminal branches of the ingroup, harming the network’s interpretation unnecessarily (as such events are not the main focus of the present study); this dataset is referred to as the 34-set. Each such LCB gene tree was estimated in IQTree following the same steps mentioned above for model selection and branch support attribution. We also employed a species tree method (ASTRAL-III) that finds the best tree by concomitantly accounting for ancestral allelic polymorphisms while being robust to moderate levels of recombination [89, 90]; all 95 genomes were included for this analysis, and LCB gene trees were estimated according to the above procedures. A species tree based on the unicopy gene trees was also estimated.

We also performed ML phylogenetic reconstruction using indel stretches as characters (based on the unicopy set), as these may reveal important phylogenetic patterns in bacteria [91]. First, we assembled a supermatrix with all the genes from the core-genome, adding a 100 bp region of in-tandem repetition of adenines (“AAA … ”) in between genes to avoid regions of gaps at the end of a gene and start of the next being grouped incorrectly as the same indel state. We input the generated concatenated MSA (plus intergenic adenines) into SeqState [41] using the “modified complex coding scheme”, which attributes numeric states to contiguous gaps that overlap across taxa in a gene, with sequences without gaps in those regions being coded as “0″. Subsequently, we recoded any states with valuer greater than or equal to 2 as state “1″, therefore treating overlapping indels as binary characters. For the ML analysis of indels, models were tested in IQTree including the “ASC” option (ascertainment bias correction, as indels lack constant sites, and the likelihood in these models must then be adjusted accordingly) [92]. ML search and branch support were calculated as described above.

### Rooting trees

The ML unicopy tree was rooted by the MAD algorithm [93], which finds the branch minimizing deviations from the midpoint criterion (i.e., the idea that assumes that the middle of the path between any two OTUs should coincide with their last common ancestor) across all possible root positions and all OTU pairs of the unrooted tree, being more accurate than other known rooting procedures [93]. This rooted unicopy ML-based tree was then fixed for recombination, dating, and biogeographic analyses. The phylograms obtained from different datasets and phylogenetic methods above were rooted by the same method.

### Population genetic analysis

Because the three pathotypes may have diverged from each other relatively recently [32] and populations may still bear high levels of mixing, two population-level analyses were conducted: (I) we used the population genetic-based BAPS v6.0 [51] with unicopy SNPs to assess the actual number of structured populations within XCC (found automatically by the software) and to infer the degree of admixture in each of them, assuming a model with linkage between SNPs; BAPS attributes individuals to populations in a Bayesian way by determining the maximal set of individuals resembling each other genetically as much as possible in each of them, while concomitantly updating the inference of the number of populations [51]; and (II) a complimentary way not assuming any model of population subdivision was also employed with the unicopy SNPs, employing DAPC in the R adegenet package [94].

### Recombination

Four recombination assessment methods were employed with LCBs. Three of them were used to detect blocks across the 95 genomes showing significant signs of recombination (PHI, NSS and MaxCHI) in the PhiPack package [95] with a significance level of 0.05. Genes bearing any significant signs of recombination were removed for a second-round ML phylogenetic reconstruction, to test for the effect of recombining regions in the estimated tree. The fourth recombination method employed was ClonalFrameML [44], to estimate the strength of recombination throughout the tree for the 34-set, considering the LCBs with at least 5000 bp (only the subset including all outgroup plus three genomes from each pathotype was employed for this analysis). Given an inferred topology, it calculates the contribution of recombination relative to single-point mutations (r/m), doing this for each branch. Kappa was fixed as the transition ratio of the transition and transversion rates obtained in the ML inference. Two ClonalFrameML runs were performed to test for convergence, each divided into two rounds: the first estimated global parameter values, then the second round applied a per-branch optimization model starting with the former global parameter values.

### Dating

Regarding dating analyses, we employed the same core LCB-based 34-taxa dataset used for network estimation. The rooted ML unicopy tree was fixed throughout most dating analyses. A test for the best molecular clock type (strict or relaxed) was carried in the R package treedater 0.2.0 [47]. Subsequently, we tested whether including tip-dating would be informative using TempEst [48]. The best clock type was then set up in BEAST v1.10.4 [49] for the remaining analyses, using as default setup (“original run”) HKY + I + G for the substitution model (easier to converge on most analyses), a birth-death prior (BD) on node times, an exponential time for the root (following bounds specified below), with rates following the literature (also mentioned below), and without removing recombining regions. We then compared the effect of different parameter/data scenarios against the original run: (I) a MCMC run without data, to test for data informativeness regarding dating (II) tree search (instead of ML-fixed topology); (III) removal of recombining regions inferred by ClonalFrameML; (IV) employing a coalescent skyline model (with five points) instead of a BD tree prior; (V) a Uniform distribution on the root age (instead of Exponential); (VI) BEAST v2.5.2 [96] to compare the effect of a different implementation of the same software; (VII) a faster overall rate of evolution (obtained from [59]); and (VIII) a more complex GTR + I + G substitution model.

Tip-dating was employed according to isolation dates in Table 1, or by assuming a uniform distribution on dates between [0, 104] ya in the case of genomes for which isolation dates were unavailable. Alternative distributions for the time to most recent common ancestor (tMRCA) of all taxa were set using 25,000 ya [32] as either a soft 95% upper bound (Exponential), or as a hard upper bound (Uniform). The minimum (hard) bound for both distribution priors was 104 ya, which corresponds to the earliest reference to *Xanthomonas citri* that we are aware of [97].

The clock rate’s hyperprior (for the strict clock’s, or for the ucld.mean parameter if the uncorrelated lognormal relaxed clock model - UCLN - was chosen) was set as uniform between 1e-09 and 1e-07 substitutions/site/branch/y (s/s/b/y), encompassing values from different sources [98–100] assuming a slowest generation time of 25 h/generation (g), and fastest being 1.1 h/g [101]. Notably, this range also encompasses rates from Mhedbi-Hajri et al. [32] for the *X. axonopodis* group based on seven housekeeping genes (of 2.0e-5 per gene/y, which for an average of 1000 bp for a *X. axonopodis* gene amounts to ~ 2.0e-8 s/s/b/y). Alternatively, a test of faster rates was employed using 1e-05 s/s/b/y as an upper bound, based on an analysis of 36 bacterial data sets by Duchêne et al. [59].

Runs with different assumptions were compared by a posterior simulation-based analog of the AIC model (AICM), because the more accurate stepping-stone procedure [102–104]) did not converge and/or induced numeric instability errors in many cases, given the 34-set alignment with 1,212,579 pb used for the dating analyses. We further note that Zarza et al. [105] showed with simulations that the performance of AICM improves substantially by using larger alignments instead of the 1000 pb datasets simulated in the papers by Baele et al. [103, 104] in which AICM is shown to be inferior to stepping stone, therefore being alignments more than 1000x smaller than the one used here. AICM values were computed as the average between the two MCMC runs for each condition tested, using Tracer v1.6 [106].

Each configuration was run twice in Tracer to avoid local optima, until putative convergence and effective sample sizes (ESSs) of parameters were ≥ 200. Highest posterior densities of 95% (HPDs) were computed using the same software. The two MCMC runs for the same set of conditions were summarized by Logcombiner (within the BEAST v1.10.4 package).

### Biogeography

Ancestral biogeographic areas were estimated for each node assuming a discrete state model of ranges employing the Bayesian Binary MCMC analysis (BBM, a method modified from Ronquist et al. [45]) in RASP [107] with two parallel chains of 100,000 steps, after recoding the 23 tip localities into more inclusive (and geographically sensible) bins whenever appropriate: Caribbean (Martinique), China, East Africa (Ethiopia, Sudan), Indian Ocean Islands (Maldives, Mauritius, Reunion, Seychelles), Indian Subcontinent (Bangladesh, India, Pakistan), Indochina (Cambodia, Thailand), Japan, Middle East (Iran, Oman, Saudi Arabia), South America (Argentina, Brazil), USA, and West Africa (Mali, Senegal, Burkina Faso), for a total of 11 areas; rate transition probabilities were considered to be equal between any two areas (JC model).

### Inferring the ancestral host

We estimated the host at internal nodes of the ML phylogeny (taking branch lengths into account) using the function ace in phytools 0.6–60 [46], which infers discrete ancestral states by empirical Bayesian posterior probabilities. Hosts at tips were defined according to Table 1, for a total of 11 different states. A model of equal rates among states was employed.

### Presence/absence analysis of pathogenicity-related genes

A set of 120 genes (63 effectors from the Xanthomonas.org site, and 57 genes previously screened for pathogenicity in pathotype A [33–38]) were analyzed by tBlastn searches [108] with e-value ≤1e-50 against the set of 95 genomes.

## Additional files

Additional file 1: **Table S1.** Genomic data associated with the six newly sequenced XCC genomes. **Table S2.** The 120 genes for which presence/absence was investigated across pathotypes: 63 effectors from the Xanthomonas.org database; and 57 pathogenicity-related genes (see text for details). (DOC 789 kb)

Additional file 2: **Figure S1.** PCA of 12 genomic features obtained by DFAST for each genome during reannotation, used to detect and remove from downstream analyses genomes that had: (1) the largest separation from the other points according to the first PC; and (2) which corresponded to worse-behaviored genomes according to any of the 12 features (e.g., less gaps; larger N50). The features considered were: Total Sequence Length (bp), Number of Sequences, Longest Sequence (bp), N50 (bp), Gap Ratio (%), GCcontent (%), Number of CDSs, Average Protein Length, Coding Ratio (%), Number of rRNAs, Number of tRNAs, and Number of CRISPRs. We found out that Average Protein Length and Coding Ratio were always smaller in the suspicious genomes, and at the same time their gap ratio was higher, suggesting these genomes could bias analyses downstream. A total of 12 genomes were eliminated. (PDF 44 kb)

Additional file 3: **Figure S2.** Saturation plots obtained in DAMBE (for transitions and transversions separately and in different colors). *x-axis:* F84-distances; *y-axis: p*-distances. (PDF 43 kb)

Additional file 4: **Figure S3.**Trees based on different datasets and/or types of analysis. Resolutions are based on branch support ≥95% (or 0.95). (PDF 672 kb)

Additional file 5: **Figure S4.** Biogegraphical ancestral area reconstruction across ingroup (XCC pathotypes) and outgroup, using the Bayesian Binary MCMC algorithm in RASP. *Areas:* (A) Caribbean; (B) China; (C) East Africa; (D) Indian Ocean Islands; (E) Indian Subcontinent; (F) Indochina; (G) Japan; (H) Middle East; (I) South America; (J) USA; (K) West Africa. (PDF 1889 kb)

Additional file 6: **Figure S5.** ProgressiveMauve alignment of the genomic island of *X. durantae* against the three plasmids from the A^w^ strain TX160149 from Texas. (PDF 32 kb)

## Data Availability

The datasets generated and/or analysed during the current study are available in the National Center for Biotechnology Information repository, as given in Table 1.

## Abbreviations

A: Pathotype A of *Xanthomonas citri* subsp. *citri*
A*: Pathotype A* of *Xanthomonas citri* subsp. *citri*
A2: Clade A2 of *Xanthomonas citri* subsp. *citri* (clustered within pathotype A)
A306: *Xanthomonas citri* subsp. *citri* strain A306
AICM: Posterior simulation-based analogue of Akaike’s information criterion
ANI: Average nucleotide identity
A^W^: Pathotype A^W^ of *Xanthomonas citri* subsp. *citri*
BBM: Bayesian Binary MCMC method
BIC: Bayesian information criterion
CI: Confidence interval
DAPC: Discriminant analysis of principal components
ESS: Effective sample size
h/g: number of hours per generation
HGT: Horizontal gene transfer
HPD: Highest posterior density
kya: thousand years ago
LCB: Locally collinear blocks
MAD: Minimum ancestor deviation method
ML: Maximum likelihood
MP: Maximum parsimony
MRCA: Most recent common ancestor
OTU: Operational taxonomic unit
PC1: First principal component
PC2: Second principal component
PCA: Principal component analysis
PE: Paired-end reads
r/m: contribution of recombination relative to single-point mutations
SE: Single-end reads
SNP: Single nucleotide polymorphism
tMRCA: time to most recent common ancestor
UCLN: Uncorrelated lognormal distribution of rates across branches
XCC: *Xanthomonas citri* subsp. *citri*
XCP: *Xanthomonas citri* pathovar
y: years
ya: years ago
