# Supplementary material for: Origin and diversification of Xanthomonas citri subsp. citri pathotypes revealed by inclusive phylogenomic, dating, and biogeographic analyses
Source: BMC Genomics. 2019 Sep 9;20:700. doi: 10.1186/s12864-019-6007-4 (PMC6734499; doi:10.1186/s12864-019-6007-4)
Supplement: Supplementary file 1 — Table S1. Genomic data associated with the six newly sequenced XCC genomes. Table S2. The 120 genes for which presence/absence was investigated across pathotypes: 63 effectors from the Xanthomonas.org database; and 57 pathogenicity-related genes (see text for details). (DOC 789 kb) [file 12864_2019_6007_MOESM1_ESM.doc]

**Suppl. Table 1:** Genomic data associated with the six newly sequenced XCC genomes.


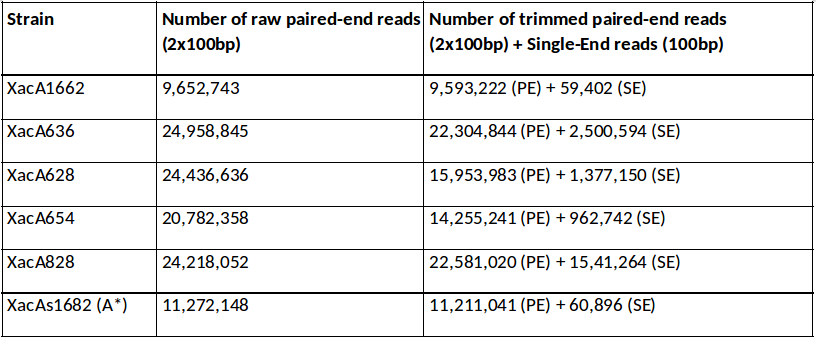


**Suppl. Table 2:** The 120 genes for which presence/absence was investigated across pathotypes: 63 effectors from the Xanthomonas.org database; and 57 pathogenicity-related genes (see text for details).

**
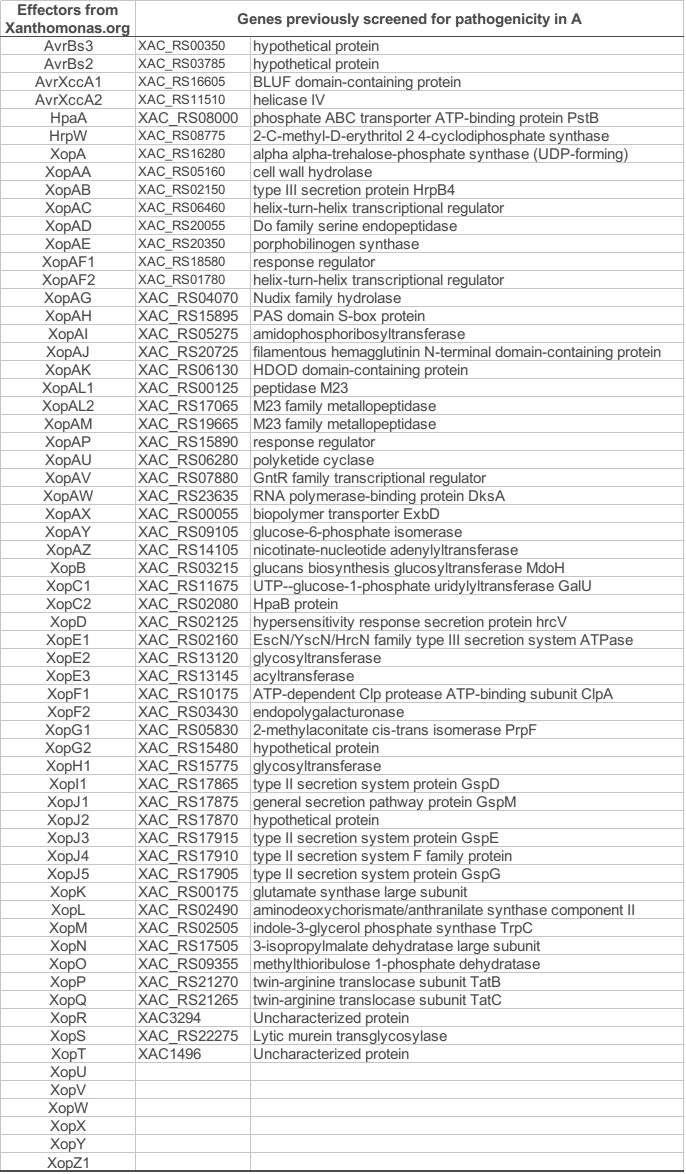
**
