# Supplementary figures and images for: Origin and diversification of Xanthomonas citri subsp. citri pathotypes revealed by inclusive phylogenomic, dating, and biogeographic analyses
Source: BMC Genomics. 2019 Sep 9;20:700. doi: 10.1186/s12864-019-6007-4 (PMC6734499; doi:10.1186/s12864-019-6007-4)

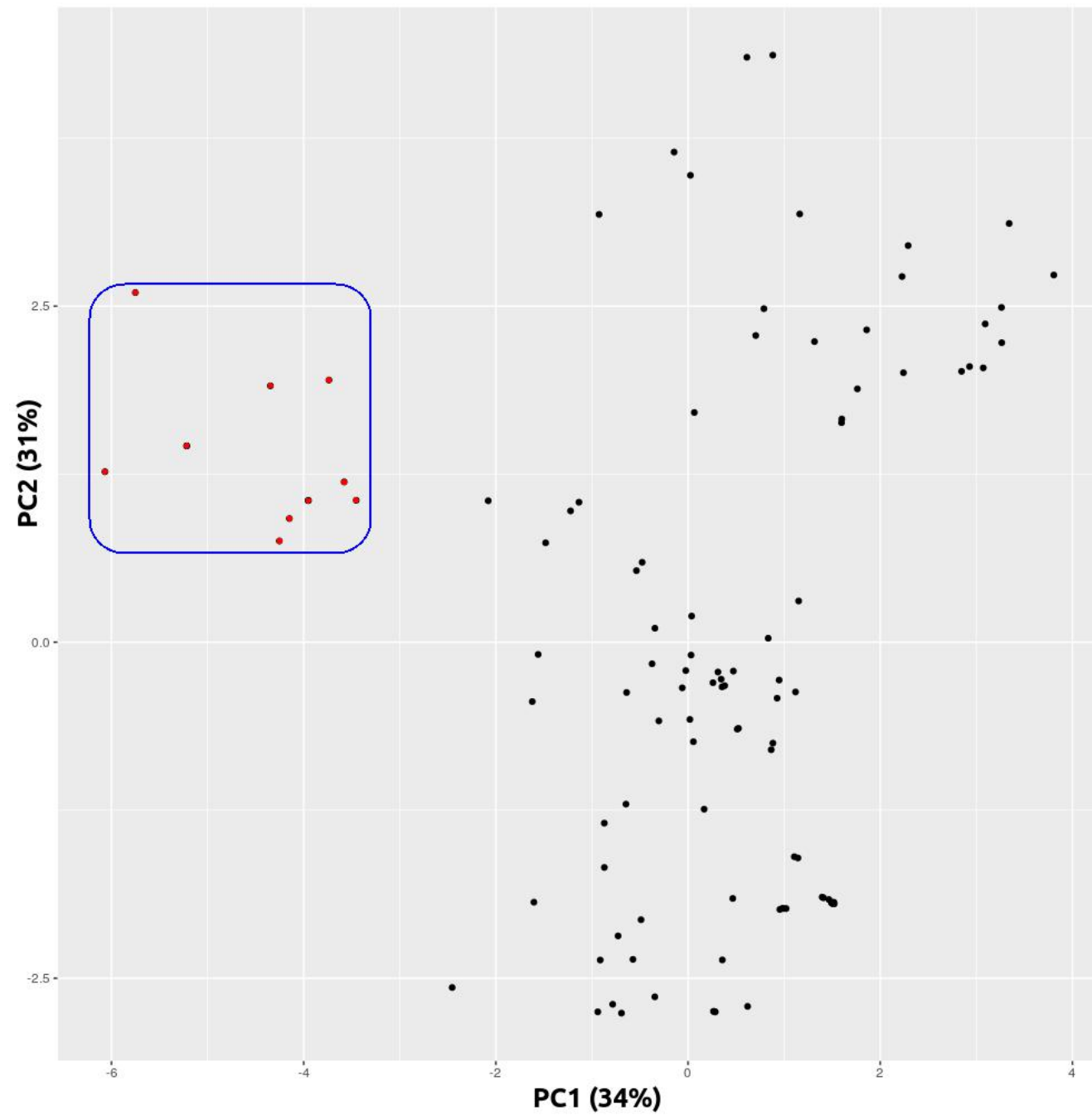

Supplement: Supplementary file 2 — Figure S1. PCA of 12 genomic features obtained by DFAST for each genome during reannotation, used to detect and remove from downstream analyses genomes that had: (1) the largest separation from the other points according to the first PC; and (2) which corresponded to worse-behaviored genomes according to any of the 12 features (e.g., less gaps; larger N50). The features considered were: Total Sequence Length (bp), Number of Sequences, Longest Sequence (bp), N50 (bp), Gap Ratio (%), GCcontent (%), Number of CDSs, Average Protein Length, Coding Ratio (%), Number of rRNAs, Number of tRNAs, and Number of CRISPRs. We found out that Average Protein Length and Coding Ratio were always smaller in the suspicious genomes, and at the same time their gap ratio was higher, suggesting these genomes could bias analyses downstream. A total of 12 genomes were eliminated. (PDF 44 kb) [file 12864_2019_6007_MOESM2_ESM.pdf]

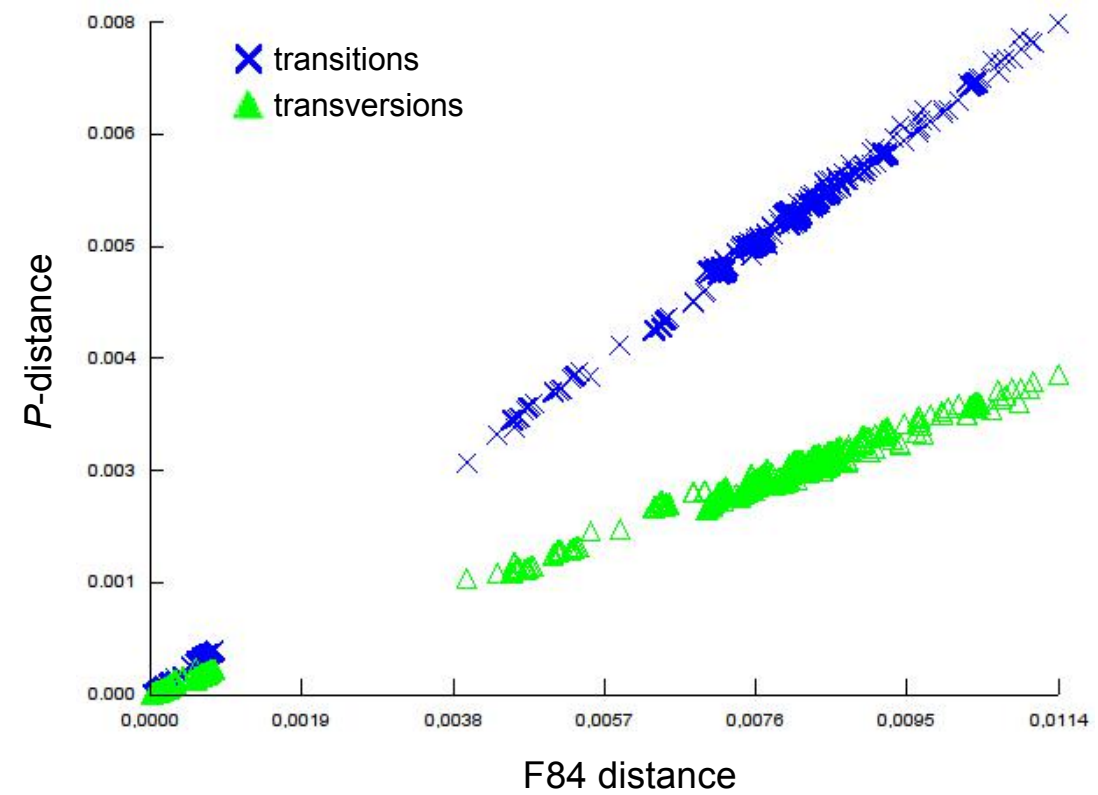

Supplement: Supplementary file 3 — Figure S2. Saturation plots obtained in DAMBE (for transitions and transversions separately and in different colors). x-axis: F84-distances; y-axis: p-distances. (PDF 43 kb) [file 12864_2019_6007_MOESM3_ESM.pdf]

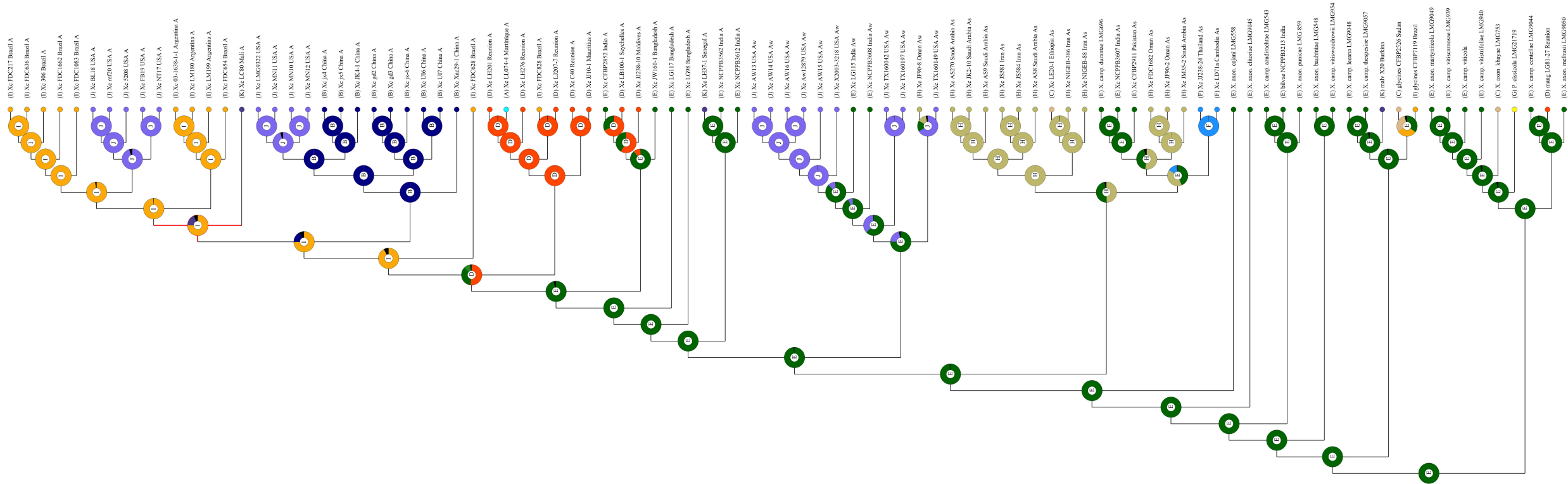

Supplement: Supplementary file 5 — Figure S4. Biogegraphical ancestral area reconstruction across ingroup (XCC pathotypes) and outgroup, using the Bayesian Binary MCMC algorithm in RASP. Areas: (A) Caribbean; (B) China; (C) East Africa; (D) Indian Ocean Islands; (E) Indian Subcontinent; (F) Indochina; (G) Japan; (H) Middle East; (I) South America; (J) USA; (K) West Africa. (PDF 1889 kb) [file 12864_2019_6007_MOESM5_ESM.pdf]

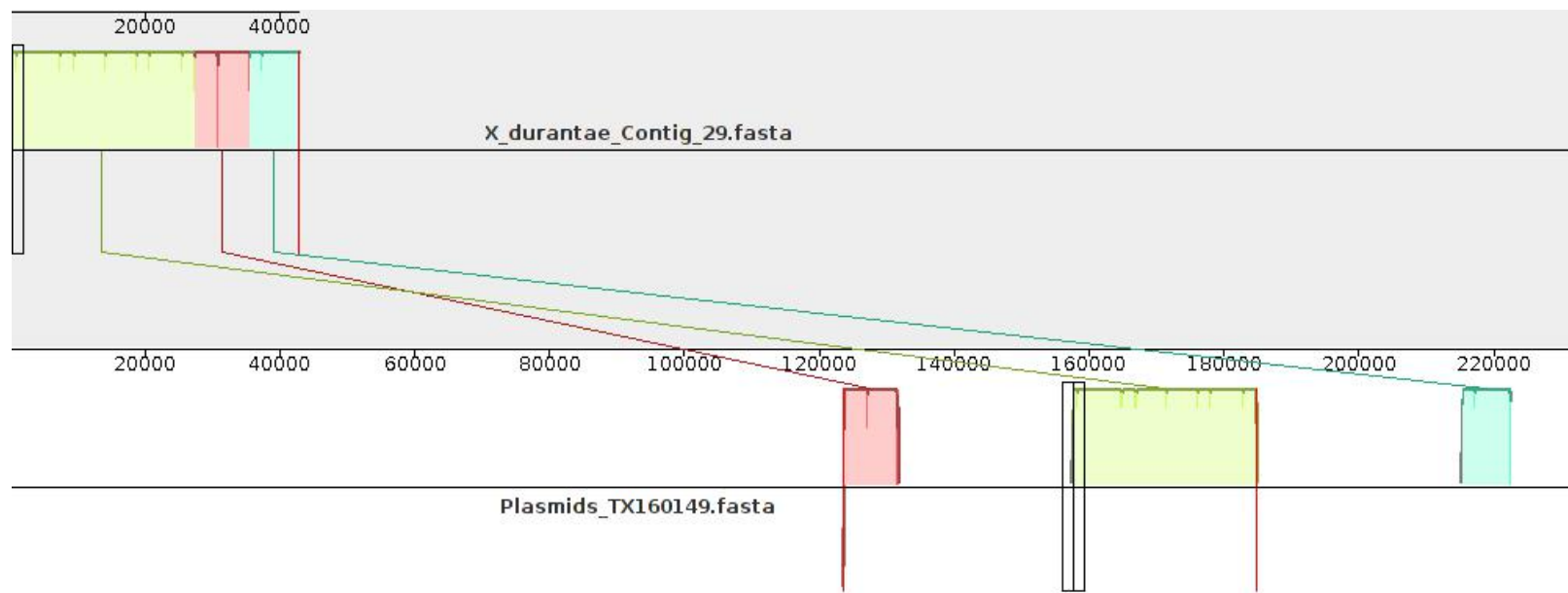

Supplement: Supplementary file 6 — Figure S5. ProgressiveMauve alignment of the genomic island of X. durantae against the three plasmids from the Aw strain TX160149 from Texas. (PDF 32 kb) [file 12864_2019_6007_MOESM6_ESM.pdf]
