# Supplementary material for: Origin and diversification of Xanthomonas citri subsp. citri pathotypes revealed by inclusive phylogenomic, dating, and biogeographic analyses
Source: BMC Genomics. 2019 Sep 9;20:700. doi: 10.1186/s12864-019-6007-4 (PMC6734499; doi:10.1186/s12864-019-6007-4)

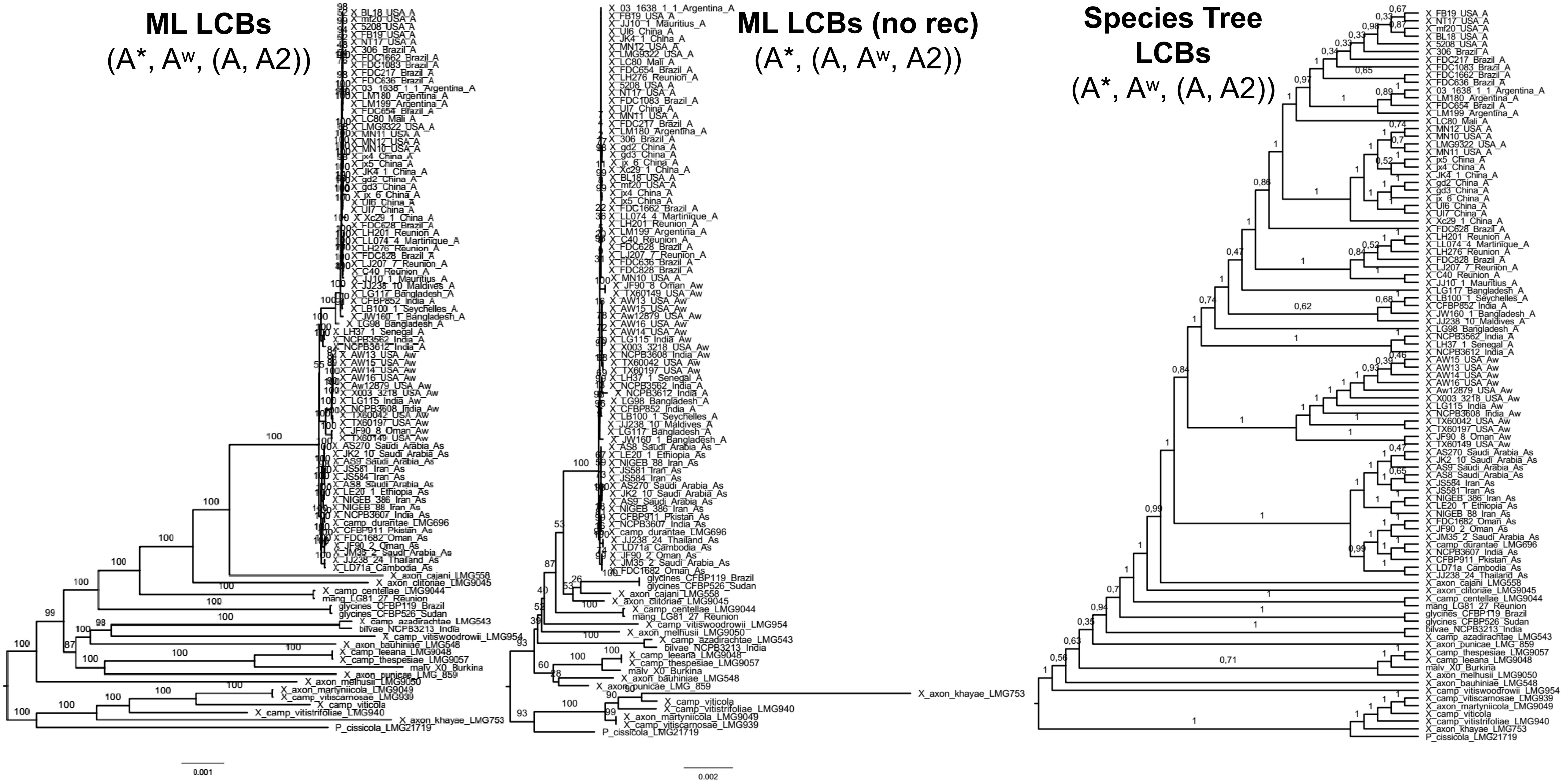
ML LCBs
(A*, Aw, (A, A2))
ML LCBs (no rec)
(A*, (A, Aw, A2))
Species Tree
LCBs
(A*, Aw, (A, A2))
0.001
0.002

ML Indels
(A2, A*, A^{w}, A)

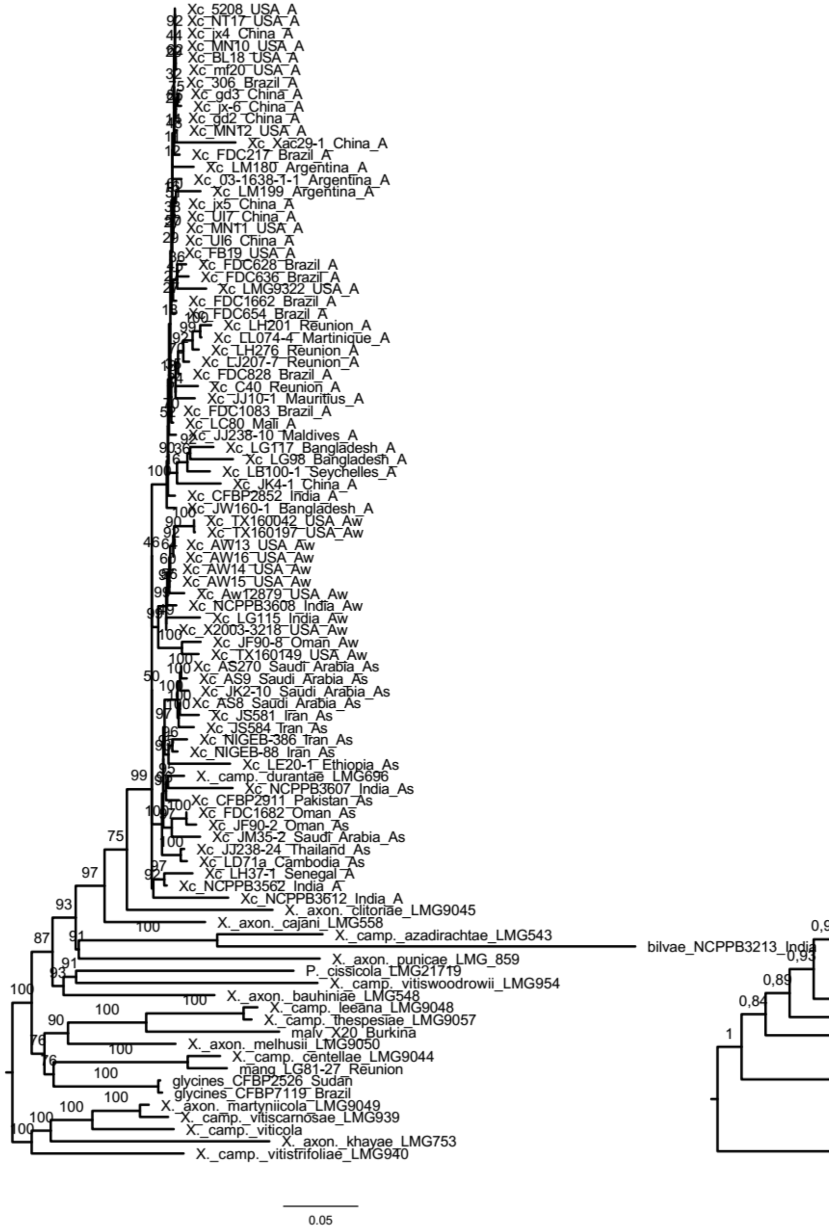

Species Tree Unicopy
((A*, A^{w}), A, A2)

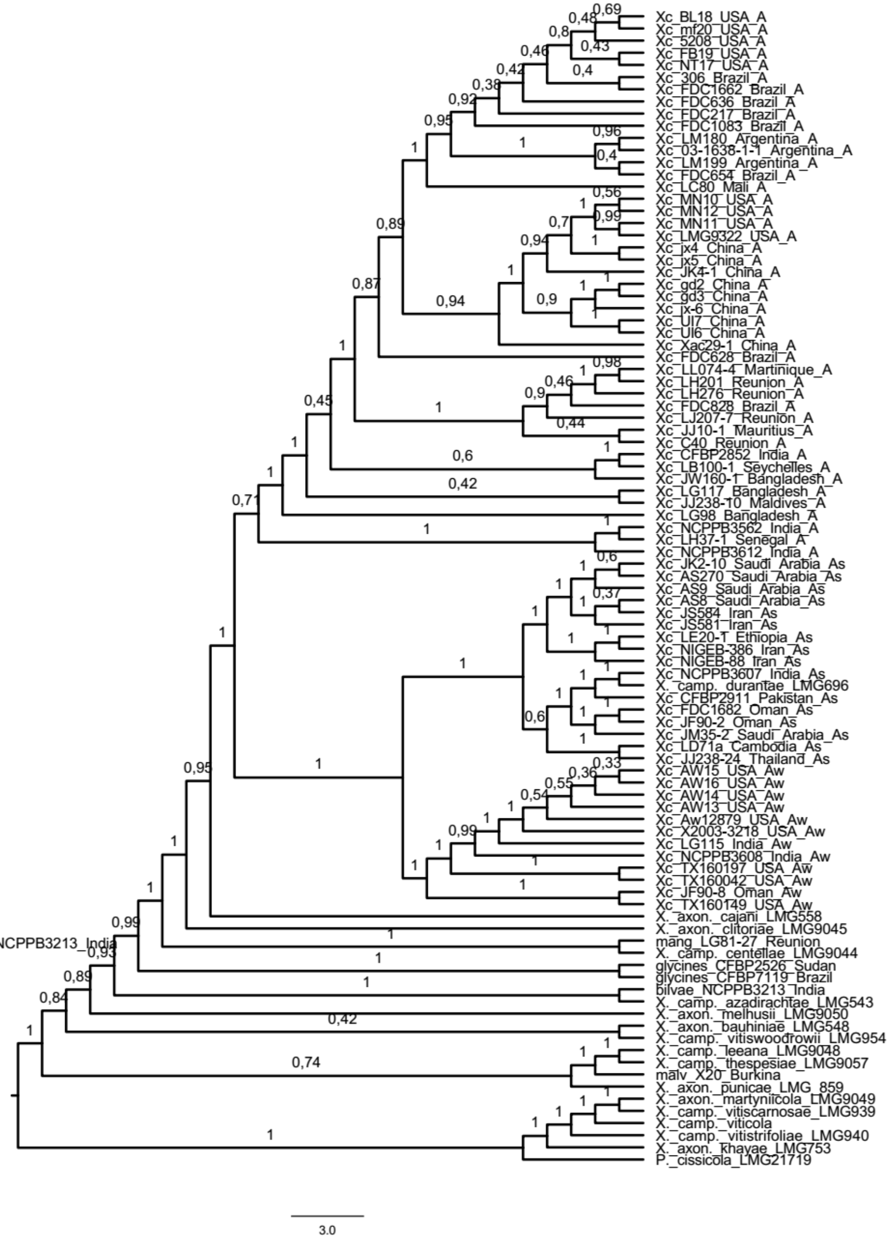

MP Unicopy
(A, (A*, (A^{w}, A2)))

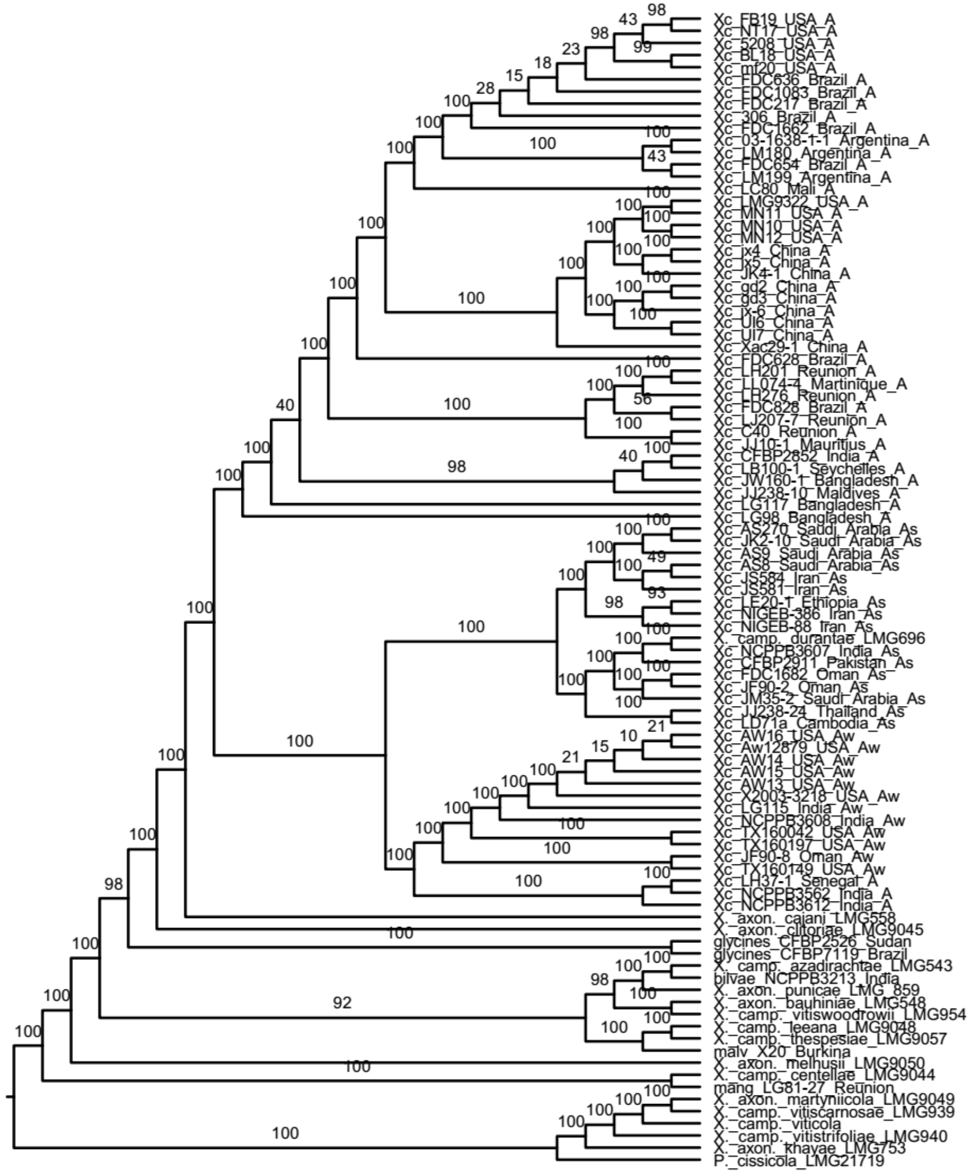

Supplement: Supplementary file 4 — Figure S3.Trees based on different datasets and/or types of analysis. Resolutions are based on branch support ≥95% (or 0.95). (PDF 672 kb) [file 12864_2019_6007_MOESM4_ESM.pdf]
